# SIRT2-mediated deacetylation and deubiquitination of C/EBPβ prevents ethanol-induced liver injury

**DOI:** 10.1038/s41421-021-00326-6

**Published:** 2021-10-12

**Authors:** Yingting Zhang, Xidai Long, Xin Ruan, Qian Wei, Lin Zhang, Lulu Wo, Dongdong Huang, Longshuai Lin, Difei Wang, Li Xia, Qinghua Zhao, Junling Liu, Qian Zhao, Ming He

**Affiliations:** 1 Department of Pathophysiology, Key Laboratory of Cell Differentiation and Apoptosis of Ministry of Education, Shanghai Jiao Tong University School of Medicine, Shanghai, China; 2 Department of Pathology, The Affiliated Hospital of Youjiang Medical University for Nationalities, Baise, Guangxi, China; 3 Department of Biochemistry and Molecular Cell Biology, Key Laboratory of Cell Differentiation and Apoptosis of the Chinese Ministry of Education, Shanghai Jiao Tong University School of Medicine, Shanghai, China; 4 Department of Orthopedics, Shanghai General Hospital, Shanghai Jiao Tong University, Shanghai, China; 5 Department of Core Facility of Basic Medical Sciences, Shanghai Jiao Tong University School of Medicine, Shanghai, China

**Keywords:** Acetylation, Stress signalling

## Abstract

Protein acetylation has emerged to play pivotal roles in alcoholic liver disease (ALD). Sirutin 2 (SIRT2) is a nicotinamide adenine dinucleotide (NAD^+^)-dependent deacetylase involved in the regulation of aging, metabolism, and stress. However, the role of SIRT2 in ALD remains unclear. Here, we report that the SIRT2-mediated deacetylation–deubiquitination switch of CCAAT/enhancer-binding protein beta (C/EBPβ) prevents ALD. Our results showed that hepatic SIRT2 protein expression was negatively correlated with the severity of alcoholic liver injury in ALD patients. Liver-specific *SIRT2* deficiency sensitized mice to ALD, whereas transgenic *SIRT2* overexpression in hepatocytes significantly prevented ethanol-induced liver injury via normalization of hepatic steatosis, lipid peroxidation, and hepatocyte apoptosis. Mechanistically, we identified C/EBPβ as a critical substrate of SIRT2 implicated in ALD. SIRT2-mediated deacetylation at lysines 102 and 211 decreased C/EBPβ ubiquitination, resulting in enhanced protein stability and subsequently increased transcription of C/EBPβ-target gene *LCN2*. Importantly, hepatic deacetylated C/EBPβ and LCN2 compensation reversed SIRT2 deletion-induced ALD aggravation in mice. Furthermore, C/EBPβ protein expression was positively correlated with SIRT2 and LCN2 expression in the livers of ALD patients and was inversely correlated with ALD development. Therefore, activating SIRT2-C/EBPβ-LCN2 signaling pathway is a potential therapy for ALD.

## Introduction

Alcoholic liver disease (ALD) is a major category of liver diseases, and the mortality from ALD continues to increase worldwide^1,2^. ALD encompasses several histopathologic changes, from simple steatosis to alcoholic steatohepatitis, progressive liver fibrosis, cirrhosis, and liver cancer^2,3^. Chronic alcohol intake promotes the accumulation of acetaldehyde and other reactive oxygen moieties in the liver, which leads to impaired hepatocyte metabolism, chronic oxidative stress, lipid peroxidation, hepatocytes damage, and death^2,4^. Although much research has investigated the pathogenesis and development of ALD, the underlying molecular mechanisms that defend against the detrimental effects of alcohol use remain elusive and current pharmacotherapy options are limited^3,5,6^. Therefore, the identification of novel therapeutic targets is an urgent clinical need in ALD.

Protein acetylation has emerged as a key posttranslational modification in alcohol metabolism^6^; yet, the regulation and mechanisms of the protein acetylation have not been completely understood in ALD. Sirtuins (SIRT) are a highly evolutionarily conserved family of nicotinamide adenine dinucleotide (NAD^+^)-dependent protein deacetylases. The seven homologs in the mammalian SIRT family (SIRT1–7) display diversity in tissue specificity, subcellular localization, enzymatic activity, and target selection^7^. SIRT1, SIRT6, and SIRT7 are mainly localized in the nucleus, where they deacetylate histones. SIRT3, SIRT4, and SIRT5 mainly localize in mitochondria^7,8^.

SIRT2 is the only sirtuin mainly located in the cytoplasm and abundantly expressed in the liver^7^. SIRT2 also may shuttle between the cytoplasm and nucleus during stress^9^. Growing evidence has suggested that SIRT2 plays an important role in the regulation of aging, glucose and lipid metabolism, cell differentiation, cell cycle, and tumorgenesis^7,8^. The previous work has demonstrated that SIRT2 maintains hepatic insulin sensitivity during physiological aging through deacetylation of NLRP3^10^. However, the role of SIRT2 in ethanol-induced liver injury has not been described.

CCAAT/enhancer-binding protein beta (C/EBPβ) is an important transcription factor involved in numerous biological processes by regulating the expression of target genes, including adipogenesis, gluconeogenic pathway, liver regeneration, hematopoiesis, and apoptosis^11^. Moreover, the activity and stability of C/EBPβ may be post-translationally regulated by various modifications including acetylation^12^. Although elevation of C/EBPβ protein in ethanol-induced hepatosteatosis in mice has been observed^13^, the role and mechanism of the dysregulation and acetylation switch of C/EBPβ in ALD are still elusive. In this study, we identify SIRT2 as a novel deacetylase of C/EBPβ and uncover a critical protective function of SIRT2-mediated deacetylation–deubiquitination switch of C/EBPβ in ethanol-induced liver injury. Our in vivo findings suggest that SIRT2-C/EBPβ signaling pathway can be potentially targeted for ALD treatment.

## Results

### SIRT2 inversely correlates with ethanol-induced liver injury in patients

**Fig. 1:**
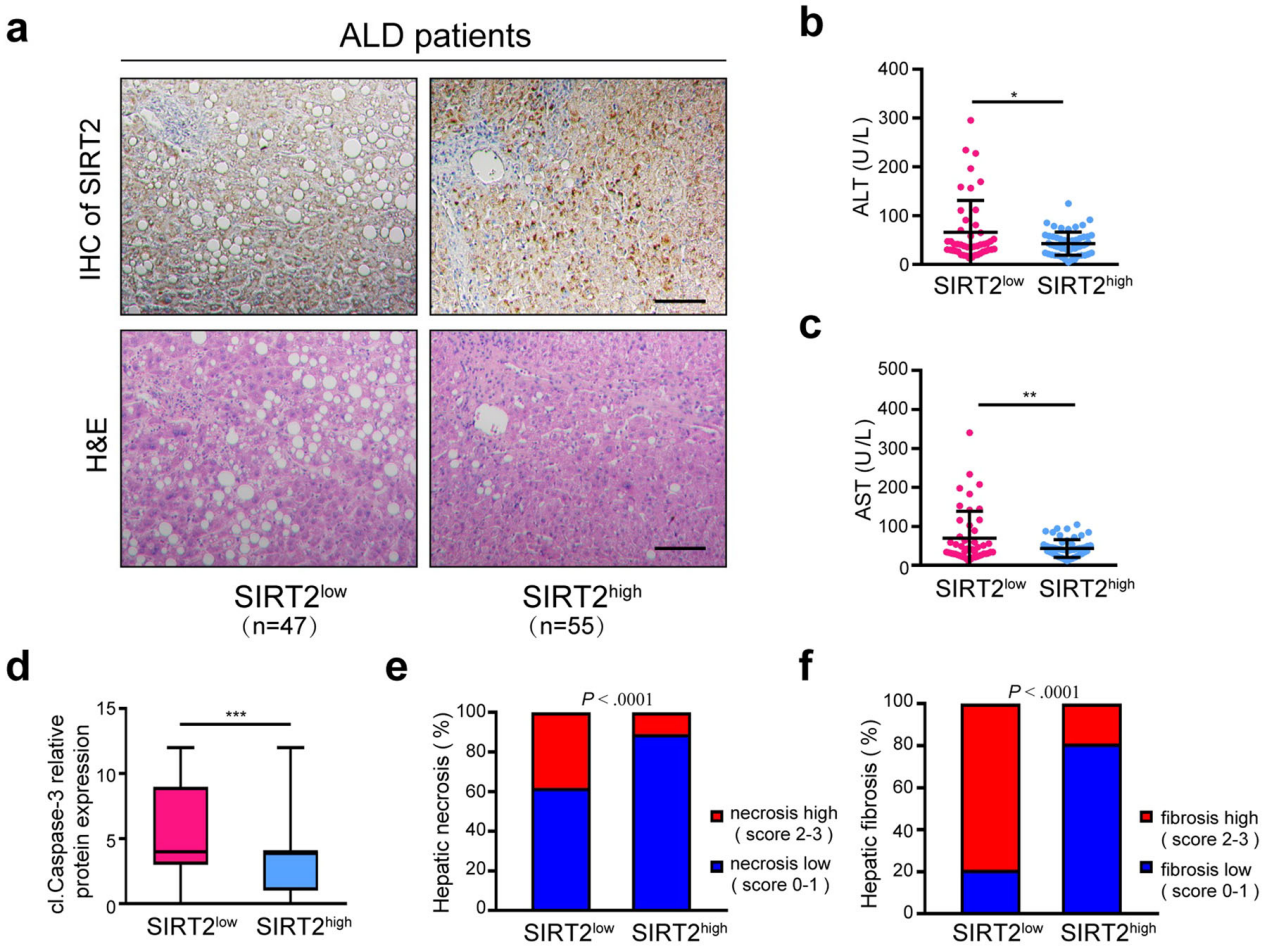
SIRT2 protein level negatively correlates with the severity of ALD in patients. **a** Representative IHC images of liver tissues from ALD patients for the lower (SIRT2^low^, IRS ≤ 5, *n* = 47) and higher (SIRT2^high^, IRS > 5, *n* = 55) expressions of SIRT2. The scale bar represents 50 μm. **b**–**f** Plots of serum ALT (**b**) and AST (**c**), cl.Caspase-3 IRS scores (**d**), and the percentages of liver tissues with high and low necrosis (**e**) or fibrosis (**f**) in SIRT2^low^ and SIRT2^high^ groups. Statistical significance was determined by two-tailed Student’s *t-*test (**b**–**d**), Pearson’s *χ*^2^ test (**e**, **f**). Data are shown as means ± SD and were considered statistically significant at *P < 0.05, **P < 0.01, and ***P < 0.001.

To assess the relevance of hepatic SIRT2 expression to alcoholic liver injury, we analyzed SIRT2 protein expression in liver samples from normal controls (*n* = 12) and patients with ALD (*n* = 102) (Supplementary Tables S1 and S2) by immunohistochemical (IHC) staining. The hepatic tissues presented different degrees of immunoreactive scores (IRS) of SIRT2, steatosis, and liver damage (Fig. 1a). The data revealed that SIRT2 protein level was significantly increased in the livers from ALD patients when compared to the normal controls through comparing the IRS of samples (Supplementary Fig. S1a). For the specimens from 102 patients, 54% of liver tissues showed higher SIRT2 expression (IRS > 5, SIRT2^high^), while 46% showed lower SIRT2 expression (IRS ≤ 5, SIRT2^low^) (Supplementary Fig. S1b). Then, we comprehensively evaluated the serum levels of alanine transaminase (ALT), aspartate transaminase (AST), and IRS of hepatic cleaved Caspase-3 (cl.Caspase-3), degree of necrosis, and fibrosis in the liver tissues of ALD patients. We found that the levels of serum ALT, AST, and hepatic cl.Caspase-3 expressions were much lower in the SIRT2^high^ group than those of the SIRT2^low^ group (Fig. 1b–d). The severity and stages of ALD may be identified by histopathologic changes. The alcoholic hepatitis histologic score (AHHS) model^14^ analysis results showed the reverse association between the expression of hepatic SIRT2 and the AHHS stage of ALD (Supplementary Fig. S1c). In addition, the liver injury score (LIS) model was used to focus on hepatic necrosis and fibrosis for making up the limitations of the AHHS model. SIRT2 protein expression was inversely correlated with ethanol-induced hepatic necrosis (Fig. 1e; Supplementary Fig. S1d) and fibrosis (Fig. 1f; Supplementary Fig. S1e). Collectively, our results showed that hepatic SIRT2 expression was negatively correlated to clinical ethanol-induced liver injury and the development of ALD.

### Liver-specific *SIRT2* deficiency sensitizes mice to alcoholic liver injury

**Fig. 2:**
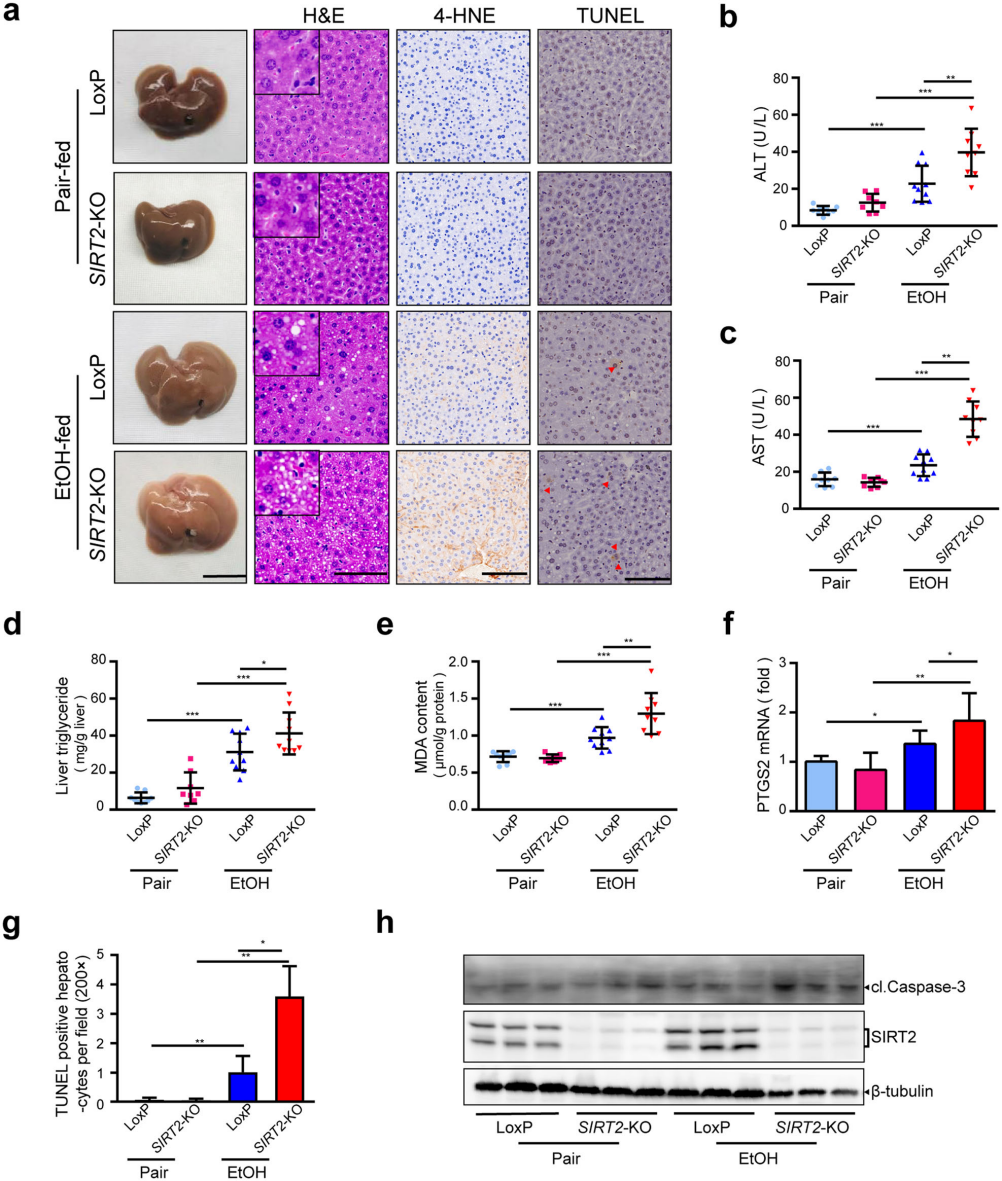
Liver-specific *SIRT2* KO sensitizes mice to alcoholic liver injury. *SIRT2*^*f/f*^*Alb-Cre*^*–*^ (LoxP) and *SIRT2*^*f/f*^*Alb-Cre*^*+*^ (*SIRT2*-KO) male mice were treated with pair (Pair) and ethanol diet (EtOH) according to NIAAA model (*n* = 8–10/group). **a**–**h** Liver injury, steatosis, lipid peroxidation, and cell apoptosis were assessed by images of the indicated livers (scale bar, 1 cm), mouse hepatic H&E staining (scale bar, 100 μm), IHC detection of 4-HNE and TUNEL (scale bar, 100 μm) (**a**), serum ALT (**b**) and AST (**c**), liver triglyceride (TG) (**d**), hepatic MDA content (**e**), and *PTGS2* mRNA (**f**), quantitative analysis of TUNEL-positive hepatocytes (magnification, ×200) (**g**), Western blot analysis of cl.Caspase-3 in murine liver tissues (**h**). Student’s *t-*test was used for statistical evaluation. Data are shown as means ± SD and are considered statistically significant at ^*^P < 0.05, ^**^P < 0.01, and ^***^P < 0.001.

To further investigate the role of hepatic SIRT2 in ALD, we generated a liver-specific *SIRT2*-deficient mice using a floxed *SIRT2* mouse strain and an Alb-Cre line (Supplementary Fig. S2a). Control *SIRT2*^*f/f*^*Alb-Cre*^*–*^ (LoxP) and *SIRT2*^*f/f*^*Alb-Cre*^*+*^ (*SIRT2*-knockout (KO)) mice were genotyped by PCR and *SIRT2* gene was efficiently deleted in the livers of *SIRT2*-KO mice (Supplementary Fig. S2b). The LoxP and *SIRT2*-KO mice were subjected to chronic-plus-binge ethanol feeding protocols (National Institute on Alcohol Abuse and Alcoholism (NIAAA) model) modified from the report by Bin Gao’s laboratory^15^. The pair-fed *SIRT2*-KO mice were phenotypically unremarkable (Fig. 2). Over the course of alcohol feeding, *SIRT2*-KO mice showed similar food intake, body weight, and organ to body weight ratios as LoxP mice (Supplementary Fig. S2c–g). However, the ethanol-fed *SIRT2*-KO mice displayed paler livers compared with the LoxP mice (Fig. 2a). Moreover, the hematoxylin–eosin (H&E) staining showed that the *SIRT2*-null livers on the ethanol diet had an increased number of vacuoles compared with those from LoxP mice (Fig. 2a), indicating more lipid accumulation. Similarly, liver triglyceride (TG) levels were significantly increased in the ethanol-fed *SIRT2*-KO mice (Fig. 2d). Meanwhile, ethanol-fed *SIRT2*-KO mice exhibited stronger lipid peroxidation in the livers as compared with ethanol-fed LoxP mice, indicated by the elevation of 4-hydroxynonenal (4-HNE) (Fig. 2a), malondialdehyde (MDA), and prostaglandin-endoperoxide synthase 2 (*PTGS2*) messenger RNA (mRNA) (Fig. 2e, f). Moreover, serum ALT and AST (Fig. 2b, c) levels, the number of terminal deoxynucleotidyl transferase dUTP nick end labeling (TUNEL)-positive hepatocytes (Fig. 2a, g), and cl.Caspase-3 expression (Fig. 2h) was markedly increased in the ethanol-fed *SIRT2*-KO mice, suggesting aggravated liver injury and apoptosis of hepatocytes. These results provide compelling evidence that liver-specific *SIRT2* deficiency exacerbates ethanol-induced TG accumulation, lipid peroxidation, and hepatocyte apoptosis.

### *SIRT2* knockdown in a hepatocyte model of ALD leads to more apoptosis and lipid peroxidation

To verify the role of SIRT2 in a cell model, we created *SIRT2* knockdown AML12 hepatocytes and assessed their response to ethanol exposure. Mouse AML12 hepatocytes efficiently metabolize ethanol and provide an adequate system to study how ethanol causes hepatocyte injury^16^. The cell count (Supplementary Fig. S3a, b), western blot analyses of Caspase-3 and PARP-1 cleavage (Supplementary Fig. S3c), and TUNEL assays (Supplementary Fig. S3d, e) demonstrated that *SIRT2* knockdown caused more ethanol-induced hepatocyte apoptosis. Meanwhile, histological analysis revealed that *SIRT2* knockdown caused an obvious elevation of ethanol-induced lipid ROS shown by C11-BODIPY and *PTGS2* mRNA expression (Supplementary Fig. S3f, g). Together, these data indicate that *SIRT2* knockdown results in increased ethanol-induced hepatocyte apoptosis with higher lipid peroxidation.

### Hepatocyte-specific AAV8-mediated *SIRT2* overexpression completely prevents ALD in mice

**Fig. 3:**
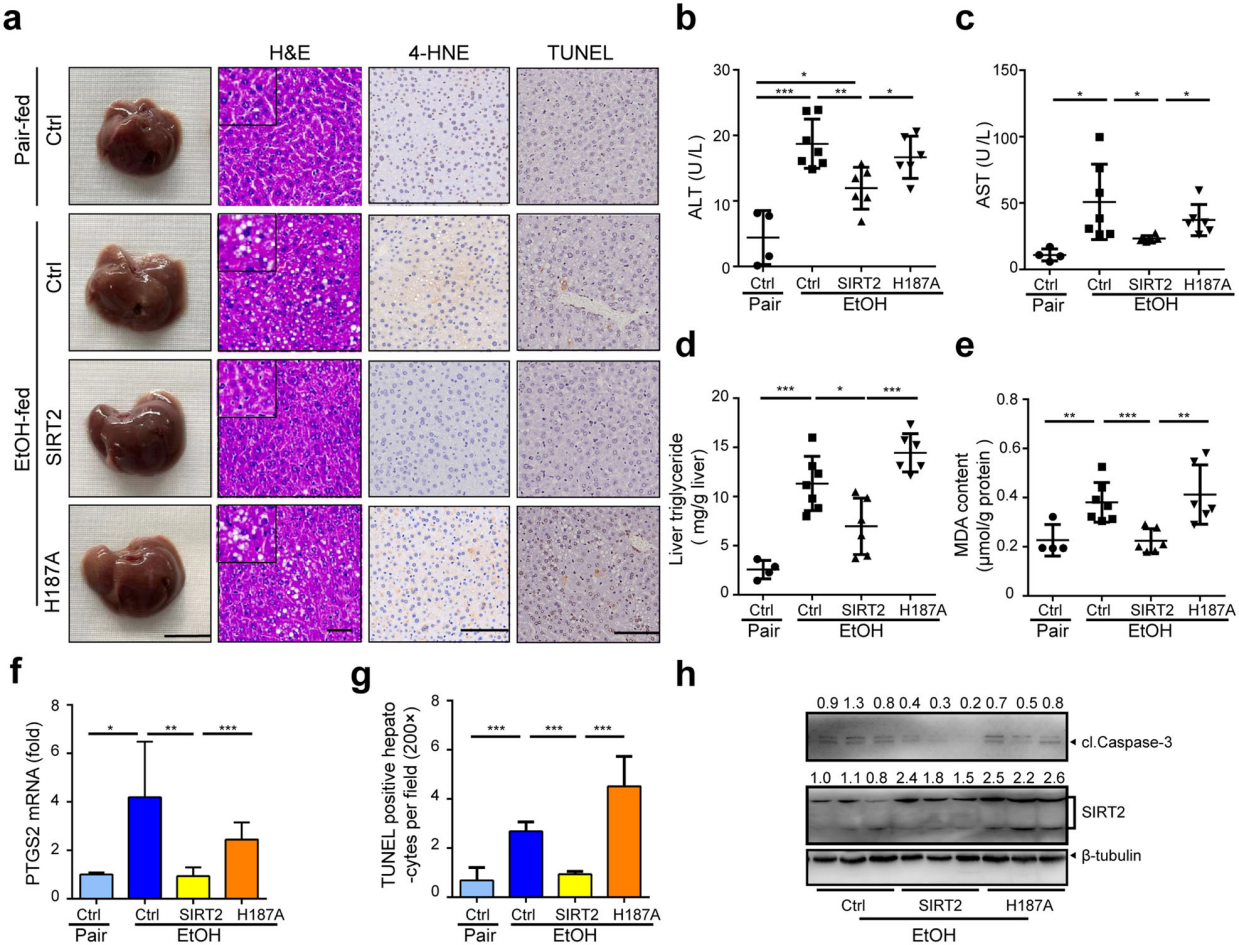
Hepatocyte-specific *SIRT2* overexpression prevents alcoholic liver injury in mice. Male C57BL/6 mice tail injected with AAV8-Ctrl (Ctrl), AAV8-SIRT2 (SIRT2), or AAV8*-*SIRT2-H187A (H187A) were treated with pair (Pair) and ethanol diet (EtOH) according to NIAAA model (*n* = 7/group). **a** Images of the indicated livers (scale bar, 1 cm), murine liver staining by H&E (scale bar, 50 μm), IHC detection of 4-HNE and TUNEL assay (scale bar, 100 μm). **b**–**d** Serum ALT (**b**) and AST (**c**) measurements and TG content (**d**). **e**–**f** Hepatic MDA measurements (**e**) and *PTGS2* mRNA analysis by qRT-PCR (**f**). **g** Quantitative analysis of TUNEL-positive hepatocytes (magnification, ×200) in murine livers. **h** Western blot analysis of cl.Caspase-3 and SIRT2 expression in murine liver tissues. Student’s *t-*test was used for statistical evaluation. Data are shown as means ± SD and are considered statistically significant at *P < 0.05, **P < 0.01, and ***P < 0.001.

To assess the potential of SIRT2 to reverse alcoholic liver injury, we developed a recombinant adeno-associated viral vector serotype 8 (AAV8) expressing *SIRT2* or its catalytic inactive *SIRT2-H187A* (H187A) mutant^17,18^ under the control of the hepatocyte-specific thyroxin-binding globulin (TBG) promoter (AAV8-SIRT2 and AAV8-H187A) (Supplementary Fig. S4a). The AAV8 vector contains a luciferase reporter gene for real-time observation of gene expression by bioluminescence imaging (BLI). We determined that the AAV8 constructs require 2 weeks for full and specific expression in the murine livers (Supplementary Fig. S4b). Therefore, we treated mice with 2 × 10^11^ viral particles containing AAV8-luciferase (Ctrl), AAV8-SIRT2, or AAV8-H187A construct via tail-vein injection 14 days before alcohol feeding (Supplementary Fig. S4c). In accordance with the BLI results (Supplementary Fig. S4d), PCR (Supplementary Fig. S4e) and western blot analysis (Fig. 3h) confirmed the hepatic SIRT2 and H187A overexpression in the AAV8-SIRT2 and AAV8-H187A groups. Hepatocyte-specific *SIRT2* overexpression totally prevented ethanol-induced increases in 4-HNE staining (Fig. 3a), serum ALT and AST (Fig. 3b, c), liver TG content (Fig. 3d), MDA content (Fig. 3e), *PTGS2* mRNA level (Fig. 3f), TUNEL-positive hepatocytes (Fig. 3a, g), and Caspase-3 cleavage (Fig. 3h), whereas H187A overexpression had no significant effects. Consistent with in vivo results, *SIRT2* overexpression, but not H187A, led to less apoptosis in AML12 hepatocytes under ethanol exposure (Supplementary Fig. S5). Together, these data suggest that overexpression of SIRT2 in hepatocytes is sufficient to prevent ethanol-induced liver injury, connecting the protective role directly to SIRT2 catalytic activity.

### SIRT2 upregulates LCN2 expression

**Fig. 4:**
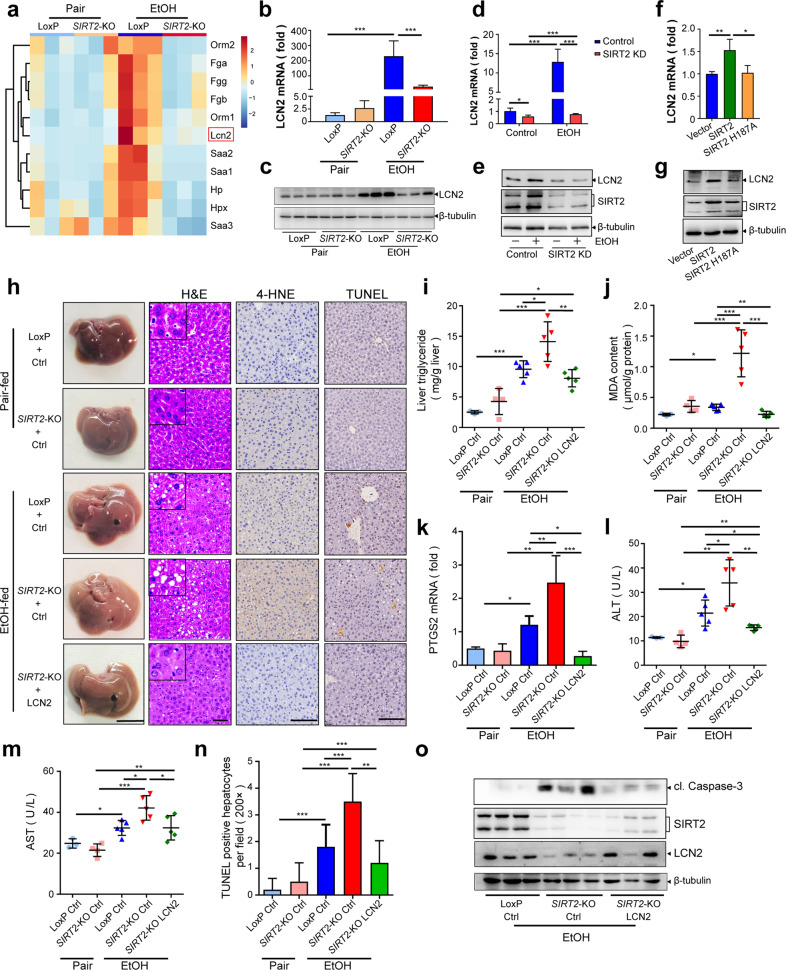
Hepatic SIRT2 protects mice from ALD through upregulating LCN2. **a** Heatmap of differentially expressed genes associated with acute-phase response (Gene Ontology: 0006953) identified by RNA-seq using liver tissues from pair- or EtOH-fed LoxP or *SIRT2*-KO mice (*n* = 3). **b**, **c** qRT-PCR (**b**) and Western blot (**c**) analyses of *LCN2* mRNA and protein expression in murine livers. **d**, **e** qRT-PCR (**d**) and Western blot analysis (**e**) of LCN2 expression in *SIRT2* knockdown (*SIRT2* KD) or control AML12 hepatocytes. **f**, **g** qRT-PCR (**f**) and Western blot analysis (**g**) of LCN2 in *SIRT2* overexpression hepatocytes. **h**–**o** LoxP and *SIRT2*-KO male mice tail-vein injected with AAV8-Ctrl (Ctrl) or AAV8-LCN2 (LCN2) were treated with pair (Pair) or ethanol diet (EtOH) according to NIAAA model construction (*n* = 3–5/group). **h** Images of the indicated livers (scale bar, 1 cm), H&E staining of murine livers (scale bar, 50 μm), IHC detection of 4-HNE and TUNEL (scale bar, 100 μm). **i**, **j** Liver TG and MDA content of indicated mice. **k** Hepatic *PTGS2* mRNA by qRT-PCR analysis of indicated mice. **l**, **m** Serum ALT and AST of indicated mice. **n** Quantitative analysis of TUNEL-positive hepatocytes (magnification, ×200). **o** Western blot analysis of cl.Caspase-3 protein in murine liver tissues. Student’s *t-*test was used for statistical evaluation. Data are shown as means ± SD and are considered statistically significant at *P < 0.05, **P < 0.01, and ***P < 0.001.

To explore how SIRT2 prevents ALD, we examined mRNA expression profiles in ALD mice livers by RNA-sequencing (RNA-seq) analysis. Altogether, 287 genes showed altered expression in 3/3 samples between livers from LoxP and *SIRT2*-KO mice with chronic-plus-binge feeding (> 2 fold, *P* < 0.05) (Supplementary Fig. S6a). Furthermore, when we subjected the RNA-seq data to gene set enrichment analysis, the top-ranked pathway affected by the *SIRT2*-KO was the acute-phase response (Supplementary Fig. S6b). Acute-phase proteins (APPs), mainly produced by hepatocytes, are the published acute-phase response signatures. Of note, RNA-seq and real-time reverse transcription-PCR (qRT-PCR) results showed the same direction of change of mRNA expression for most APPs (*Fga*, *Fgg*, *Fgb*, *Orm1*, lipocaline-2 (*LCN2*), *Saa2*, *Saa1*, *Hp*, *Hpx*, *Orm2*, *and Saa3*), in which *SIRT2* deletion significantly suppressed ethanol-induced upregulation of APPs, even to the levels of pair-fed groups (Fig. 4a; Supplementary Fig. S6c). Among the most significantly regulated APPs, the previous studies reported that LCN2 functions as an antioxidant and plays an important role in protecting against liver injury^19–22^; therefore, we focused on whether LCN2 was regulated by SIRT2 in ALD. Consistent with in vivo results (Fig. 4b, c), *SIRT2* knockdown significantly inhibited ethanol-induced elevation of LCN2 expression in AML12 hepatocytes (Fig. 4d, e). Meanwhile, wild-type SIRT2, but not its catalytically inactive H187A mutant, upregulated *LCN2* mRNA and protein (Fig. 4f, g), connecting LCN2 expression directly to SIRT2 catalytic activity.

### Hepatocyte-specific *LCN2* overexpression completely prevents ALD in mice

To clarify the role of hepatocyte-expressing LCN2 in ALD, LoxP mice were given injections of either hepatocyte-specific expressed TBG-AAV8-LCN2 (LCN2) or AAV8-luciferase (Ctrl) prior to NIAAA alcohol construction. As shown in Supplementary Fig. S7, hepatocyte-specific *LCN2* overexpression completely reversed ethanol-induced increases in the liver to body weight ratio (Supplementary Fig. S7b), TG accumulation (Supplementary Fig. S7c), lipid peroxidation (Supplementary Fig. S7a, d, e), serum ALT and AST (Supplementary Fig. S7f, g), and hepatocytes apoptosis (Supplementary Fig. S7a, h). Together, hepatic LCN2 plays a protective role in alcoholic liver injury.

### SIRT2 protects against alcoholic liver injury by upregulating LCN2

To determine whether the protective effect of SIRT2 on ALD is mediated by upregulation of LCN2, LoxP and *SIRT2*-KO mice were given injections of either AAV8-luciferase (Ctrl) or AAV8-LCN2 (LCN2) prior to chronic-plus-binge feeding. The reverse of LCN2 expression was verified to be comparable to endogenous levels by western blot (Fig. 4o). Restoration of LCN2 expression in hepatocytes completely rescued the *SIRT2* deletion-induced ALD aggravation, including liver TG accumulation (Fig. 4i), serum ALT and AST levels (Fig. 4l, m), lipid peroxidation (Fig. 4h, j, k), and hepatocyte apoptosis (Fig. 4h, n, o). Together, these results suggest that the aggravated alcoholic liver injury in *SIRT2*-KO mice is, to a large extent, a consequence of downregulated LCN2 expression in hepatocytes.

### SIRT2 upregulates LCN2 through C/EBPβ under ethanol stress

**Fig. 5:**
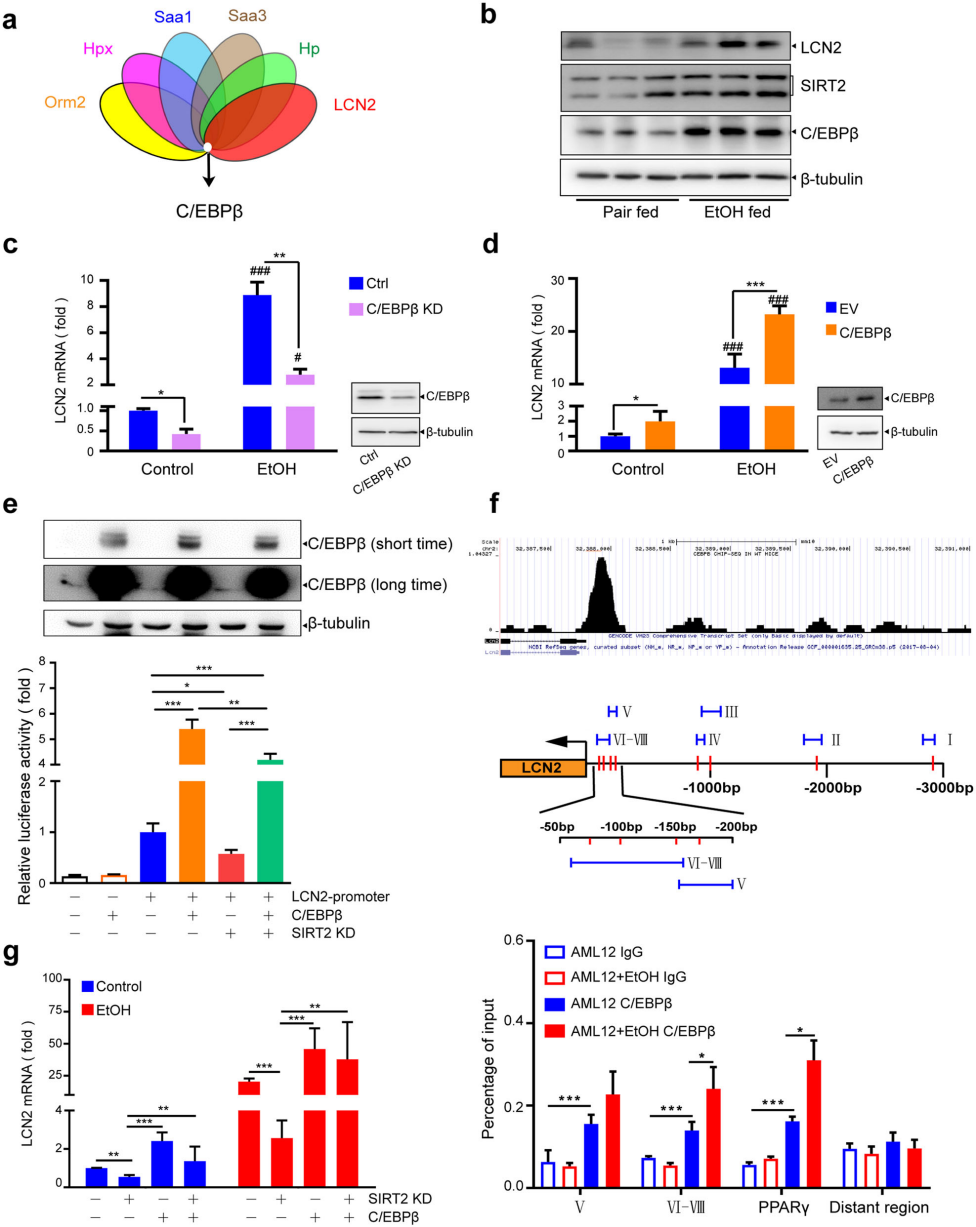
C/EBPβ mediates the upregulation of LCN2 by SIRT2 under ethanol stress. **a** Venn diagram shows common transcription factors predicted as the putative shared regulators on LCN2, Hp, Saa3, Saa1, Hpx, and Orm2. **b** Western blot analysis of hepatic LCN2, C/EBPβ, and SIRT2 expression in pair and EtOH-fed mice. **c**, **d** *LCN2* mRNA expression after *C/EBPβ* knockdown (KD) or overexpression (C/EBPβ) in AML12 cells by qRT-PCR analysis. ^#^*P* < 0.05 and ^###^*P* < 0.001 are used to indicate statistical significance compared between the group with EtOH treatment and the corresponding group without EtOH treatment. **e** Dual-luciferase reporter assay was performed in HK293T cells. Western blot analysis of C/EBPβ expression (top) and luciferase activities of the LCN2 promoter-reporter system (bottom) are shown. **f** UCSC Epigenome Browser tracks of the C/EBPβ ChIP-seq signal −3 kb before TSS of LCN2 from Cistrome DB ToolKit (top); information about predicted C/EBPβ binding sites and the primers targeting different sites on LCN2 promoter (middle); ChIP analysis showing C/EBPβ occupancy at the LCN2 proximal promoter in AML12 cells treated with EtOH and control (bottom). PPARγ and distant region primers were, respectively, used as a positive and negative control. **g** qRT-PCR analysis of *LCN2* mRNA expression in *SIRT2* knockdown AML12 cells transfected with C/EBPβ. Student’s *t*-test was used for s*t*atistical evaluation. Data are shown as means ± SD and are considered statistically significant at *P < 0.05, **P < 0.01, and ***P < 0.001.

Next, we addressed the mechanism by which LCN2 transcription is upregulated by SIRT2. Previous reports have shown that SIRT2 may deacetylate various transcription factors^7,8^. Therefore, we hypothesize that SIRT2 may regulate LCN2 transcription by affecting the expression or activity of certain transcription factors. Using the *Match program* (version 1.0), C/EBPβ was predicted as the only shared transcription factor on the six most significant SIRT2-regulated APPs (Fig. 5a). C/EBPβ protein was significantly upregulated in the livers of ethanol-fed mice, which paralleled the increased expression of LCN2 and SIRT2 (Fig. 5b). Furthermore, C/EBPβ positively regulated *LCN2* mRNA expression in AML12 hepatocytes (Fig. 5c, d). To further validate the direct regulation of C/EBPβ on LCN2 transcription, we performed dual-luciferase reporter assay in HK293T and AML12 cells. C/EBPβ overexpression significantly increased the relative luciferase activity of the LCN2 promoter and rescued *SIRT2* knockdown-caused suppression of luciferase activity both in HEK293T (Fig. 5e) and AML12 cells (Supplementary Fig. S8a). Then, according to previously reported chromatin immunoprecipitation-seq (ChIP-seq) data^23^ and via *PROMO* analysis (Fig. 5f, top), we predicted eight C/EBPβ binding sites at the LCN2 proximal promoter and designed six primers encompassing all binding sites (Fig. 5f, middle). ChIP assays showed that the fragments containing the sixth to eighth predicted C/EBPβ binding sites were mainly immunoprecipitated by anti-C/EBPβ antibodies and ethanol promoted the recruitment, indicating that −150 to ~−50 bp is the main binding region for C/EBPβ and mediated the upregulation of LCN2 by ethanol (Fig. 5f, bottom; Supplementary Fig. S8b). Furthermore, *C/EBPβ* or *SIRT2* overexpression cannot increase the relative luciferase activity of LCN2 T-150 promoter truncation, in which −150 to −1 bp was deleted from the 3-kb LCN2 promoter (Supplementary Fig. S8c). The exogenous C/EBPβ overexpression reversed the suppression of *LCN2* mRNA expression caused by *SIRT2* knockdown under the ethanol exposure (Fig. 5g; Supplementary Fig. S8d). These results identified C/EBPβ as a novel transcription factor regulating LCN2 by directly binding to specific motifs in its promoter, which mediates the upregulation of LCN2 by SIRT2 under ethanol stress.

### SIRT2 deacetylates C/EBPβ

**Fig. 6:**
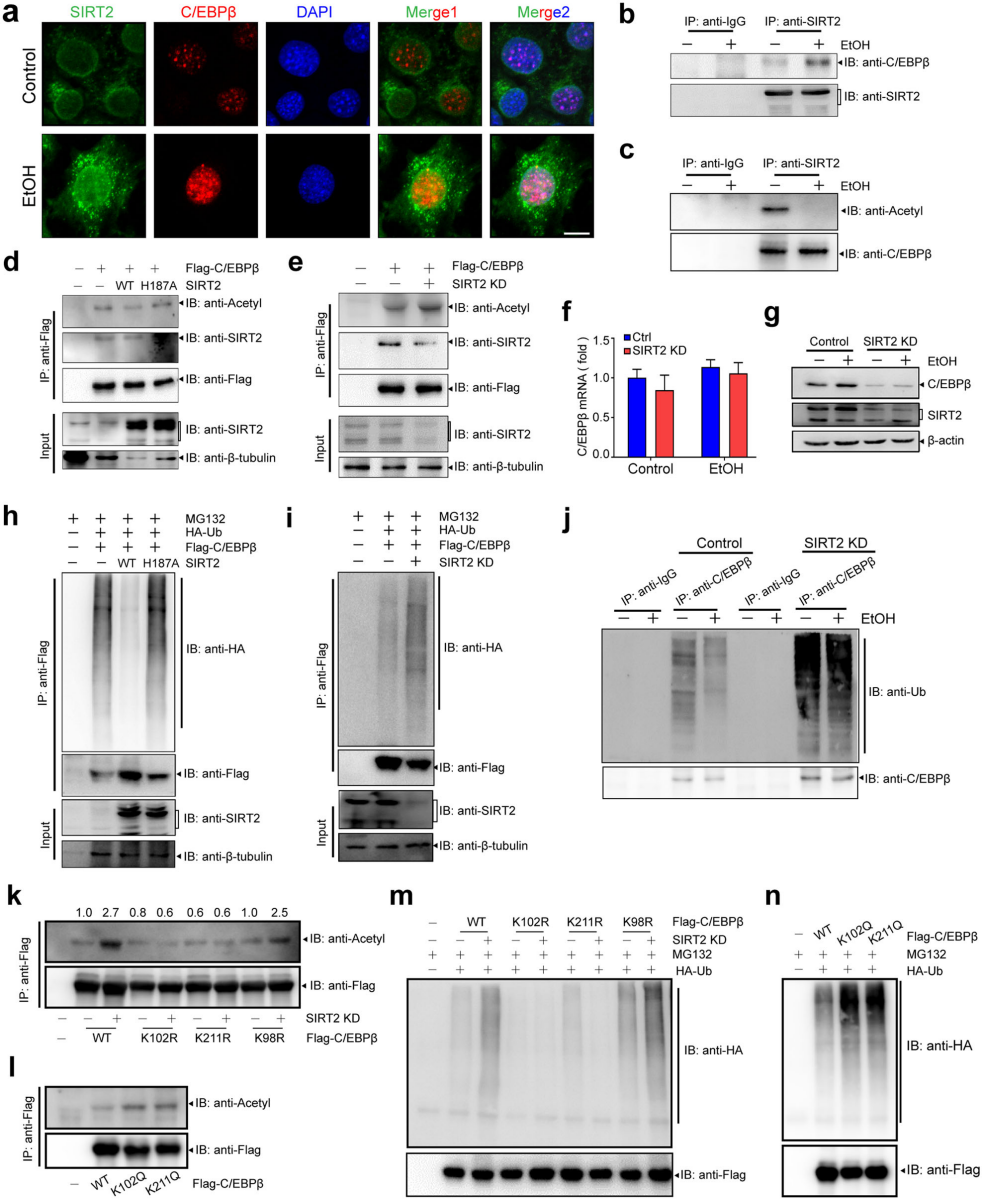
SIRT2 decreases C/EBPβ ubiquitination and stabilizes C/EBPβ protein by deacetylation under ethanol stress. **a** Immunofluorescence analysis of SIRT2 and C/EBPβ in AML12 cells treated with EtOH (scale bar, 50 μm). **b** Endogenous SIRT2–C/EBPβ interaction was analyzed by the amount of C/EBPβ coimmunoprecipitated with the same loading amount of SIRT2 in AML12 cells. **c** Acetylation levels of the same loading amount of endogenous C/EBPβ purified by IP in ethanol-treated AML12 cells detected by pan-acetyllysine antibody are shown, which indicates SIRT2 deacetylates C/EBPβ. **d**, **e** Acetylation levels of IP-purified Flag-C/EBPβ in HEK293T cells coexpressing SIRT2, SIRT2-H187A (**d**) or with *SIRT2* knockdown (**e**). **f**, **g** qRT-PCR (**f**) and Western blot analysis (**g**) of C/EBPβ expression in *SIRT2* knockdown or control AML12 cells treated with or without ethanol. **h**, **i** HEK293T cells co-transfected Flag-tagged C/EBPβ with the indicated plasmids or siRNA were treated with 10 μM MG132 or DMSO for 6 h. Ubiquitination level of C/EBPβ was probed by anti-HA antibody. **j** Ubiquitination levels of the same loading amount endogenous C/EBPβ purified by IP were probed by pan-ubiquitin antibody. **k** Acetylation levels of IP-purified C/EBPβ and its mutants in HEK293T cells with *SIRT2* knockdown. K98R is used as a negative control. **l** Acetylation levels of IP-purified Flag-C/EBPβ and its mutants in HEK293T cells. **m** Ubiquitination levels of IP-purified Flag-C/EBPβ and its mutants in HEK293T cells with *SIRT2* knockdown. **n** Ubiquitination levels of IP-purified Flag-C/EBPβ and its mutants in HEK293T cells. The experiments were repeated at least for three times with the same results, and the results of one representative experiment are shown.

Since LCN2 mediates the protective role of SIRT2 and SIRT2 upregulates LCN2 through C/EBPβ in ALD, we investigated the mechanism by which SIRT2 regulates C/EBPβ. Immunofluorescence staining and western blot results showed that ethanol significantly increased the protein expression and nuclear colocalization of SIRT2 and C/EBPβ (Fig. 6a; Supplementary Fig. S9a). Meanwhile, the immunoprecipitation assay further validated that ethanol significantly enhanced endogenous interaction of C/EBPβ with SIRT2 (Fig. 6b) and suppressed endogenous C/EBPβ acetylation in AML12 hepatocytes (Fig. 6c). Moreover, coexpressing wild-type SIRT2, but not its catalytically inactive H187A mutant, with C/EBPβ caused a decrease of C/EBPβ acetylation level (Fig. 6d), connecting C/EBPβ deacetylation directly to SIRT2 catalytic activity. In contrast, Flag-C/EBPβ acetylation level was increased after endogenous *SIRT2* was knocked down in AML12 cells (Fig. 6e). Of note, consistent with in vitro results, SIRT2, but not H187A mutant, inhibited the endogenous acetylation level of C/EBPβ in the livers of ALD mice (Supplementary Fig. S11a, b). Collectively, these data suggest that SIRT2 is a deacetylase of C/EBPβ.

### SIRT2 decreases C/EBPβ ubiquitination and stabilizes C/EBPβ protein under ethanol stress

Acetylation has been identified as an evolutionarily conserved modification in proteins and plays important roles in protein stability^24^. Notably, *SIRT2* knockdown had no effect on C/EBPβ transcription, but significantly decreased the C/EBPβ protein level in ethanol-treated AML12 cells and liver tissues (Fig. 6f, g; Supplementary Fig. S9b, c), suggesting that SIRT2 regulates C/EBPβ at posttranscriptional level. Both SIRT2 small interfering RNA (siRNA) and its inhibitor thiomyristoyl remarkably promoted C/EBPβ degradation upon cycloheximide treatment (Supplementary Fig. S10a, b). Furthermore, proteasome inhibitor MG132 treatment, but not the lysosome inhibitor chloroquine, completely reversed the downregulation of C/EBPβ protein by *SIRT2* knockdown or inhibition (Supplementary Fig. S10c–f), suggesting that SIRT2 might stabilize C/EBPβ protein through inhibiting proteasome-mediated degradation. Consistent with this notion, wild-type SIRT2, but not H187A mutant, significantly decreased C/EBPβ ubiquitination (Fig. 6h), whereas *SIRT2* knockdown or deficiency significantly enhanced C/EBPβ ubiquitination levels in vitro (Fig. 6i) and in vivo (Supplementary Fig. S11c, d). Importantly, *SIRT2* deletion significantly rescued the ethanol-suppressed endogenous C/EBPβ ubiquitination level (Fig. 6j). These observations indicate that SIRT2-mediated deacetylation decreases C/EBPβ ubiquitination and enhances its protein stability under ethanol stress.

### SIRT2 inhibits C/EBPβ ubiquitination by deacetylating lysines 102 and 211

To identify the C/EBPβ acetylation sites regulated by SIRT2, we transfected Flag-C/EBPβ with SIRT2 siRNA in HEK293T cells and then performed mass spectrometry (MS) analysis. MS analysis on purified C/EBPβ revealed that two lysine residues (K102/K211) were highly acetylated after *SIRT2* knockdown (Supplementary Fig. S12a, b). A genomic analysis demonstrated that K102 and K211 are highly conserved among different species throughout evolution (Supplementary Fig. S12c). We further generated four single mutations by replacing K102 or K211 of C/EBPβ with an arginine (R) or a glutamine (Q), respectively, and analyzed the acetylation of these C/EBPβ mutants by SIRT2. The K to R mutation mimics the deacetylated state, whereas the K to Q mutation abolishes the positive charge and may act as a surrogate of acetylation^25^. Our results showed that either the K102R or K211R mutation completely reversed the enhancement of C/EBPβ acetylation and ubiquitination level caused by *SIRT2* knockdown (Fig. 6k, m). Moreover, K102Q and K211Q mutants showed enhanced acetylation as compared with wild-type C/EBPβ in the presence of endogenous SIRT2 (Fig. 6l, n). These results indicate that acetylation of K102 and K211 mediates the regulation of SIRT2 on C/EBPβ protein stability.

### C/EBPβ deacetylation reverses *SIRT2* deletion-induced ALD aggravation

**Fig. 7:**
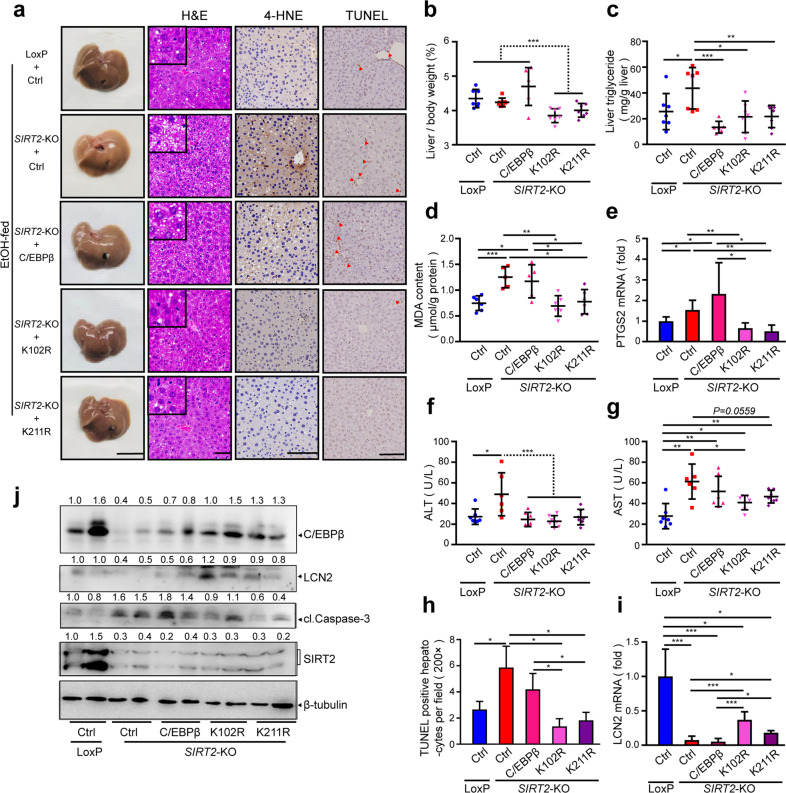
C/EBPβ deacetylation reverses hepatic *SIRT2* deficiency-aggravated ALD in mice. LoxP and *SIRT2*-KO mice tail injected with AAV8-Ctrl (Ctrl) or AAV8*-*C/EBPβ (C/EBPβ) or AAV8-C/EBPβ K102R (K102R) or AAV8-C/EBPβ K211R (K211R) were treated with NIAAA model (*n* = 7/group). **a**–**h** The effects of wild-type C/EBPβ or constitutively deacetylated C/EBPβ mutants on steatosis, lipid peroxidation, and cell apoptosis were assessed by images of the indicated livers (scale bar, 1 cm), hepatic H&E staining (scale bar, 50 μm), IHC detection of 4-HNE and TUNEL (scale bar, 100 μm) (**a**), liver/body weight ratios (**b**), liver TG (**c**), hepatic MDA content (**d**), and *PTGS2* mRNA (**e**), serum ALT (**f**), and AST (**g**), quantitative analysis of TUNEL-positive hepatocytes (magnification, ×200) (**h**). **i** Hepatic *LCN2* mRNA analysis by qRT-PCR. **j** Western blot analysis of cl.Caspase-3, LCN2, C/EBPβ, and SIRT2 protein expression in the indicated livers. Student’s *t*-test was used for statistical evaluation. Data are shown as means ± SD and are considered statistically significant at *P < 0.05, **P < 0.01, and ***P < 0.001.

To determine whether the C/EBPβ acetylation switch in hepatocytes contributes to the protective role of SIRT2 against ALD in vivo, we treated ethanol-fed LoxP and *SIRT2*-KO mice with AAV8-C/EBPβ (C/EBPβ), AAV8-C/EBPβ K102R (K102R), and AAV8-C/EBPβ K211R (K211R) or AAV8-luciferase (Ctrl) viral particles via tail-vein injection. The five ethanol-fed groups showed similar food intake and body weight. Notably, K102R and K211R mutants reversed *SIRT2* deficiency-aggravated alcoholic liver injury, including elevated liver to body weight ratio (Fig. 7b), TG accumulation (Fig. 7c), lipid peroxidation (Fig. 7a, d, e), serum ALT and AST level (Fig. 7f, g), and hepatocyte apoptosis (Fig. 7a, h, j). However, wild-type C/EBPβ had no effect on the enhancement of the lipid peroxidation (Fig. 7a, d, e) and hepatocyte apoptosis (Fig. 7a, h, j) caused by *SIRT2* deletion. Consistent with the results in AML12 cells, K102R or K211R mutant protein level was significantly higher than that of wild-type C/EBPβ in *SIRT2*-KO murine livers (Fig. 7j), whereas the viral infection and transcription level were similarly indicated by BLI and qRT-PCR (data not shown). Importantly, this reversal of alcoholic liver injury was not due to non-physiological levels of C/EBPβ overexpression, because the reconstituted expression of K102R or K211R was comparable to endogenous C/EBPβ levels in ethanol-fed LoxP group (Fig. 7j). Moreover, both mRNA and protein level of target gene *LCN2* paralleled the protein expression of C/EBPβ and mutants (Fig. 7i, j), which partially explained the different protective roles in ALD between wild-type C/EBPβ and K102R/K211R. Together, these data suggest that SIRT2-mediated C/EBPβ deacetylation and protein stability is directly linked to ethanol-induced liver injury in vivo.

### C/EBPβ inversely correlates with ALD and positively correlates with SIRT2 and LCN2 expression in patients

**Fig. 8:**
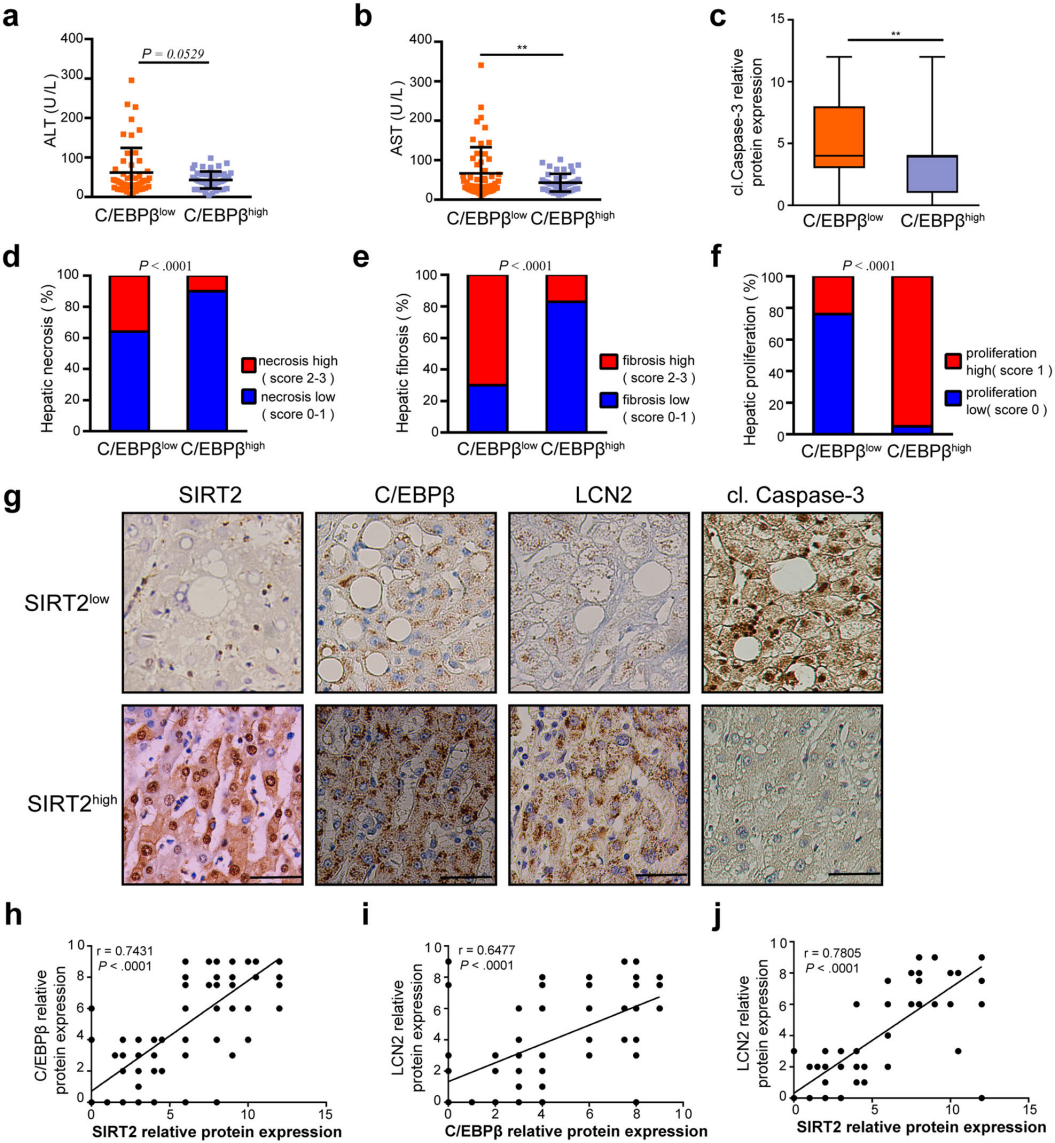
Hepatic C/EBPβ negatively correlates with alcoholic liver injury and positively correlates with SIRT2 and LCN2 expression in patient livers. **a**–**f** Plots of serum ALT (**a**) and AST (**b**), cl.Caspase-3 IRS scores (**c**), the percentages of liver tissues with different degrees of necrosis (**d**), fibrosis (**e**), and proliferation (**f**) in ALD patients with high (C/EBPβ^high^, *n* = 48) and low (C/EBPβ^low^, *n* = 54) C/EBPβ expressions. **g** Representative IHC images of patient liver samples for the low and high expressions of indicated proteins (scale bar, 100 μm). **h**–**j** Correlation analysis of relative protein expression of SIRT2, C/EBPβ, and LCN2 in liver tissues from ALD patients (*n* = 102). Statistical significance was determined by two-tailed Student’s *t* test (**a**–**c**), Pearson’s *χ*^2^ test (**d**–**f**), and linear correlation and regression (**h**–**j**). Data are shown as means ± SD and are considered statistically significant at **P < 0.01.

To further validate the relevance of hepatic C/EBPβ protein expression to ALD, we assessed C/EBPβ levels in liver tissues from 102 patients with ALD by IHC. The results showed that serum levels of ALT, AST, and hepatic cl.Caspase-3 expression were much lower in the C/EBPβ^high^ group than those in the C/EBPβ^low^ group (Fig. 8a–c). Moreover, C/EBPβ protein expression was inversely correlated with ethanol-induced hepatic necrosis and fibrosis (Fig. 8d, e; Supplementary Fig. S13a, b), while being positively correlated with hepatic proliferation (Fig. 8f; Supplementary Fig. S13c). Furthermore, consistent with the results from mice and AML12 hepatocytes, SIRT2, C/EBPβ, and LCN2 protein expressions were positively correlated with each other in the livers of ALD patients (Fig. 8g–j). Collectively, our results suggest that SIRT2-C/EBPβ-LCN2 axis suppresses clinical alcoholic liver injury.

## Discussion

The current study uncovers a critical protective role of SIRT2-mediated deacetylation of C/EBPβ against the ethanol-induced liver injury using patient specimens, along with animal and cell models. Mechanistically, we revealed for the first time that SIRT2 inhibits ubiquitination and degradation of C/EBPβ by deacetylation, and subsequently promotes the transcription of LCN2 under the ethanol stress. Importantly, this regulatory mechanism can be targeted to reverse the ethanol-induced liver injury. These findings establish SIRT2-C/EBPβ-LCN2 as a crucial axis protecting against ethanol-induced liver injury.

The liver is the major organ involved in alcohol degradation, oxidizing alcohol to acetaldehyde via alcohol dehydrogenase, and cytochrome P450 2E1 (CYP 2E1). In addition to the direct toxic effects of acetaldehyde production, chronic heavy alcohol consumption substantially upregulates and activates CYP2E1, which is associated with high production of reactive oxygen species (ROS). ROS attacks various subcellular organelles by initiating lipid peroxidation, with the generation of the products such as 4-HNE and MDA^6^. These lipid peroxidation products then lead to a chain reaction and liver injury. The role of SIRT2 in the modulation of oxidative stress through its different deacetylation targets is controversial. On the one hand, SIRT2 suppresses oxidative stress or increases antioxidant production and protects against cell apoptosis through deacetylating FOXO3a, PGC-1α, G6PD, NRF2, c-MYC, PGAM2, or regulating Mfn2, Drp1, and TFAM in mitochondria^26,27^. On the other hand, SIRT2 promotes oxidative stress-induced cell death through deacetylating PRDX-1, JNK, and S6K1^27–29^. The methodologies in these previous studies did not allow for consideration of the differences caused by various stimulations and potential interaction among different organs in *SIRT2*-KO mice. In the present study, we used liver-specific KO mice and AAV8 viral expression system with hepatocyte-specific TBG promoter to investigate the role of hepatic SIRT2 in ALD. The current measurements of 4-HNE, MDA, and PTGS2 strongly suggest the protective roles of hepatic SIRT2-C/EBPβ-LCN2 axis against ethanol-induced oxidative stress and lipid peroxidation. Meanwhile, data were presented to show that *SIRT2* mRNA and protein expression were elevated by ethanol. A previous study has reported that H_2_O_2_ or menadione does not change the transcriptional expression of SIRT2^25^. Therefore, whether ethanol upregulates the SIRT2 transcription-independent of oxidative stress remains to be explored.

Accumulating previous studies reported that SIRT2 plays different roles in the fibrosis of various organs, including cardiac fibrosis, liver fibrosis, pancreatic fibrosis, pulmonary fibrosis, and renal fibrosis. On the one hand, SIRT2 acts as a protective deacetylase in cardiac fibrosis and SIRT2 deficiency exacerbates aging-related and angiotensin II (Ang II)-induced cardiac fibrosis through AMPK-LKB1 and NFAT signaling^30,31^. Moreover, the decreased expression of SIRT2 is associated with lipopolysaccharide or doxorubicin-induced cardiac fibrosis^32,33^. For the pancreas, *SIRT2*-KO induces extensive pancreatic fibrosis in the caerulein-induced pancreatitis mouse model^34^. On the other hand, a pro-fibrotic action of SIRT2 has been documented in renal and pulmonary fibrosis. Inhibition of SIRT2 alleviates renal tubulointerstitial fibrosis in the mouse model of obstructive nephropathy^35,36^ and pulmonary fibrosis in the ovalbumin-induced allergic airway inflammation murine model^37^. However, there are conflicting reports on the roles of SIRT2 in liver fibrosis. Li et al. reported that SIRT2 mediates the protective role of NAD^+^-boosting therapy in high-fat diet-induced fibrosis by deacetylation of Fndc5^38^. On the contrary, in carbon tetrachloride- and thioacetamide-induced fibrosis mouse models, SIRT2 inhibition represses fibrogenic gene expression in hepatic stellate cells and prevents the development of hepatic fibrosis^39^. Therefore, the role of SIRT2 in fibrosis not only relates to the tissue specialty but also relates to the properties of the stimuli. In this study, the results from liver tissues of ALD patients display that hepatic SIRT2 was negatively associated with alcoholic liver fibrosis. Because NIAAA mice do not develop liver fibrosis, the more ideal mouse model may be helpful to further identify the roles of SIRT2 in alcoholic liver fibrosis.

In addition, there are various types of liver-resident immune cells^40^ and the pathogenesis and development of ALD are closely related to inflammation^41^. Our previous work demonstrated that SIRT2 in macrophages maintains hepatic insulin sensitivity through deacetylating NLRP3^10^. However, the RNA-seq results showed that hepatocyte-specific *SIRT2*-KO had no impact on the alcohol-induced inflammation (Supplementary Fig. S6), which is further verified by similar infiltration of neutrophils and macrophages, phosphorylation of NF-κB-p65, and expression of IL-1β and MCP1 in the EtOH-fed livers of *SIRT2*-KO and LoxP mice (Supplementary Fig. S14).

Protein acetylation plays important roles in protein stability and has been reported to be involved in alcohol metabolism and ALD^6^. Here, using MS analysis and site-directed mutagenesis, we identified C/EBPβ as a novel and critical target of SIRT2 implicated in ALD. Moreover, unlike the regulation of transcriptional activity by acetylation^42^, the current data have revealed for the first time that acetylation switch may regulate C/EBPβ protein ubiquitination and stability. Our present work does not preclude the role of SIRT2 in the regulation of C/EBPβ transcriptional activation, but suggests a novel mechanism of regulating C/EBPβ protein stability at the posttranslation level. Moreover, K102R/K211R mutants, not wild-type C/EBPβ, may completely reverse the SIRT2 deletion-aggravated alcoholic liver injury by stabilizing C/EBPβ, supporting that SIRT2-mediated deacetylation-deubiquitination ‘switch’ is crucial for C/EBPβ to prevent ALD.

Our results showed that the polyubiquitination level of the K102R mutant was decreased compared with the WT C/EBPβ (Fig. 6m), suggesting the possibility that K102 is one of the ubiquitination sites. To further preclude that possibility, we mutated K to Q and found that K102Q mutation does not eliminate the polyubiquitination level, but elevates both ubiquitination and acetylation of C/EBPβ, suggesting that K102 is not the direct polyubiquitination site (Fig. 6l, n). C/EBPβ is a labile protein and is tightly controlled by polyubiquitination mediated by the E3 ubiquitin ligases Mdm2 and Nrdp1, and the ubiquitin-modifying enzyme A20^11^. The mechanisms by which SIRT2-mediated deacetylation regulates C/EBPβ polyubiquitination, including the identification of the ubiquitination sites of C/EBPβ, need further study.

LCN2 is an APP and a stress-responsive molecule that protects against various stresses. Accumulating previous studies reported that LCN2 functions as an antioxidant and plays an important role in protecting against liver injury^19–22^. On the other hand, Cai et al. reported that *LCN2*-KO mice were protected from ALD by reducing hepatic steatosis, liver injury, and neutrophil infiltration compared to WT controls^43^. However, in the same report, the authors indicated paradoxically that the protective phenotype observed in *LCN2*-KO mice was not seen in hepatocyte-specific *LCN2*-KO mice after ethanol administration. Given that LCN2 is secreted by a number of cell types, such as hepatocytes, neutrophils, adipocytes, and endothelia, the previous reports did not allow for consideration of the potential interaction among different organs in *LCN2*-KO mice^44,45^. In the current work, we have revealed for the first time the regulatory mechanism and protective roles of hepatocyte LCN2 in ALD using TBG-promoter AAV8 virus (Fig. 4; Supplementary Fig. S7). Meanwhile, we note that WT C/EBPβ did not promote the expression of *LCN2* in alcoholic *SIRT2*-KO liver, but it was still able to reduce liver TG and serum ALT level (Fig. 7). This indicates that LCN2 is partially responsible for C/EBPβ acetylation-mediated phenotypes. The other targets of C/EBPβ in ALD need to be further studied in the future.

In the current study, we used the AHHS and LIS system to identify the correlation between hepatic SIRT2 and the severity of ALD. Although the results of the two analyses demonstrate an obvious reverse association between the expression of hepatic SIRT2 and liver injury/stage of ALD, the other scoring systems (e.g., MELD^46^) did not show the correlation. This may be because of the small sample size and selective bias from the hospital-based recruitment of only patients with the pathological test. Therefore, the patient sample size needs to be expanded in the future to comprehensively analyze the correlation between hepatic SIRT2-C/EBPβ-LCN2 axis and the liver injury in different types of ALD patients.

**Fig. 9:**
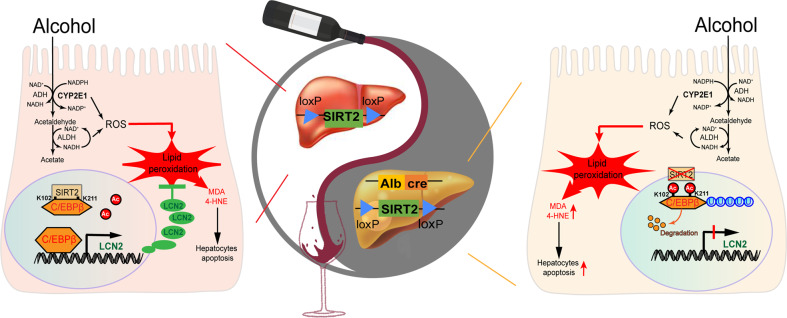
Model. Protective mechanism of hepatic SIRT2-C/EBPβ-LCN2 axis in ALD. Ethanol upregulated SIRT2 in hepatocytes, which directly deacetylates C/EBPβ on lysines 102 and 211 and inhibits ubiquitination of C/EBPβ, and subsequently promotes the transcription of LCN2. This in turn prevents oxidative stress and lipid peroxidation induced by ethanol. *SIRT2* KO increases ubiquitination and degradation of C/EBPβ and aggravates ethanol-induced liver injury.

In conclusion, we have provided comprehensive evidence showing that hepatic SIRT2 stabilizes C/EBPβ protein via direct deacetylation at K102/K211, thereby promoting the expression of LCN2. This in turn prevents hepatic steatosis, lipid peroxidation, and hepatocyte apoptosis caused by ethanol consumption, and subsequently protects the mice against ethanol-induced liver injury. Conversely, deficiency in SIRT2 promotes the ubiquitination and degradation of C/EBPβ protein and aggravates ALD (shown in Model in Fig. 9). The inverse correlation between SIRT2-C/EBPβ-LCN2 axis expression and alcoholic liver injury in clinical samples further strengthen this novel mechanism. Remarkably, hepatocyte-specific reconstitution of LCN2 or deacetylated C/EBPβ mutants with AAV8 may completely reverse the *SIRT2*-KO mice phenotype with ethanol-fed mice. Moreover, hepatocyte-specific overexpression of LCN2 or SIRT2 with AAV8 may effectively protect mice against alcoholic liver injury. Therefore, future therapeutic interventions targeting the SIRT2-C/EBPβ-LCN2 signaling pathway may serve as a potential therapy for ALD.

## Materials and methods

### Human study

Study subjects, including 102 patients with ALD and 12 controls without any evidence of liver diseases with paraffin-embedded hepatic biopsy tissues, were collected from the affiliated hospitals of Youjiang Medical University for Nationalities between January 2006 and December 2015. ALD was diagnosed based on the history of alcohol drinking and biopsy-diagnosed hepatic injury. Before samples were collected, we obtained Institutional Review Board approval. Demographic and clinicopathological information for all subjects, including gender, age, race, drinking and smoking information, and hepatic function, were obtained through the patients’ medical records, and detailed information is described in Supplementary Tables S1 and S2. This study was approved by the ethics committees of the participating hospitals and was carried out in accordance with the approved guidelines.

### Animal models

The liver-specific *SIRT2*-KO mice were produced by crossing *SIRT2*^*flox*/*flox*^ mice obtained from Johan Auwerxd Laboratory (Switzerland)^47^ and Alb-Cre mice purchased from Jackson Laboratory (USA) in a C57BL/6 background. Recombinant AAV8 constructs expressing targeted mouse *SIRT2*, *SIRT2-H187A*, *LCN2*, *C/EBPβ*, or mutated *C/EBPβ* (*K101R* and *K102R*) under the control of TBG promoter were generated by Sunbio Techservice Inc. (Shanghai, China). A noncoding plasmid carrying only the TBG promoter was used to produce control vector particles. All animals received care in compliance with protocols approved by the Institutional Animal Use and Care Committee of Shanghai Jiaotong University School of Medicine.

### Statistical analysis

All the statistical analyses were performed by GraphPad Prism 7.02 (GraphPad Software Inc. La Jolla, CA). The Pearson’s *χ*^2^ test was used to evaluate the correlation between SIRT2 or C/EBPβ expression and hepatic necrosis, fibrosis, or proliferation degree. The correlations of relative protein expression of SIRT2, C/EBPβ, and LCN2 were analyzed using linear correlation and regression. Comparisons of the two groups were analyzed using the unpaired two-tailed Student’s *t*-test. For data with more than two groups, a one-way analysis of variance with Tukey’s multiple-comparison test was used. Data are shown as means ± standard deviation (SD) and are considered statistically significant at *P < 0.05, **P < 0.01, and ***P < 0.001. All experiments were repeated at least three times with the same results, and the results of one representative experiment are shown.

Additional methods can be found in Supplementary Data S1.

### Supplementary information

Supplementary Data S1

Supplementary information
