# Author Correction: SIRT2-mediated deacetylation and deubiquitination of C/EBPβ prevents ethanol-induced liver injury

**DOI:** 10.1038/s41421-023-00618-z

**Published:** 2023-11-03

**Authors:** Yingting Zhang, Xidai Long, Xin Ruan, Qian Wei, Lin Zhang, Lulu Wo, Dongdong Huang, Longshuai Lin, Difei Wang, Li Xia, Qinghua Zhao, Junling Liu, Qian Zhao, Ming He

**Affiliations:** 1 Department of Pathophysiology, Key Laboratory of Cell Differentiation and Apoptosis of Ministry of Education, Shanghai Jiao Tong University School of Medicine, Shanghai, China; 2 Department of Pathology, The Affiliated Hospital of Youjiang Medical University for Nationalities, Baise, Guangxi China; 3 Department of Biochemistry and Molecular Cell Biology, Key Laboratory of Cell Differentiation and Apoptosis of the Chinese Ministry of Education, Shanghai Jiao Tong University School of Medicine, Shanghai, China; 4 Department of Orthopedics, Shanghai General Hospital, Shanghai Jiao Tong University, Shanghai, China; 5 Department of Core Facility of Basic Medical Sciences, Shanghai Jiao Tong University School of Medicine, Shanghai, China

Correction to: *Cell Discovery* (2021) 7:93

10.1038/s41421-021-00326-6 published online 12 October 2021

In the original publication of this article, we inadvertently misplaced an incorrect image for the 4-HNE (Pair-fed LoxP group) in Fig. 2a. The correct Fig. 2a is displayed as below. This correction does not affect the results or the conclusion of this work.

**Fig. 2:**
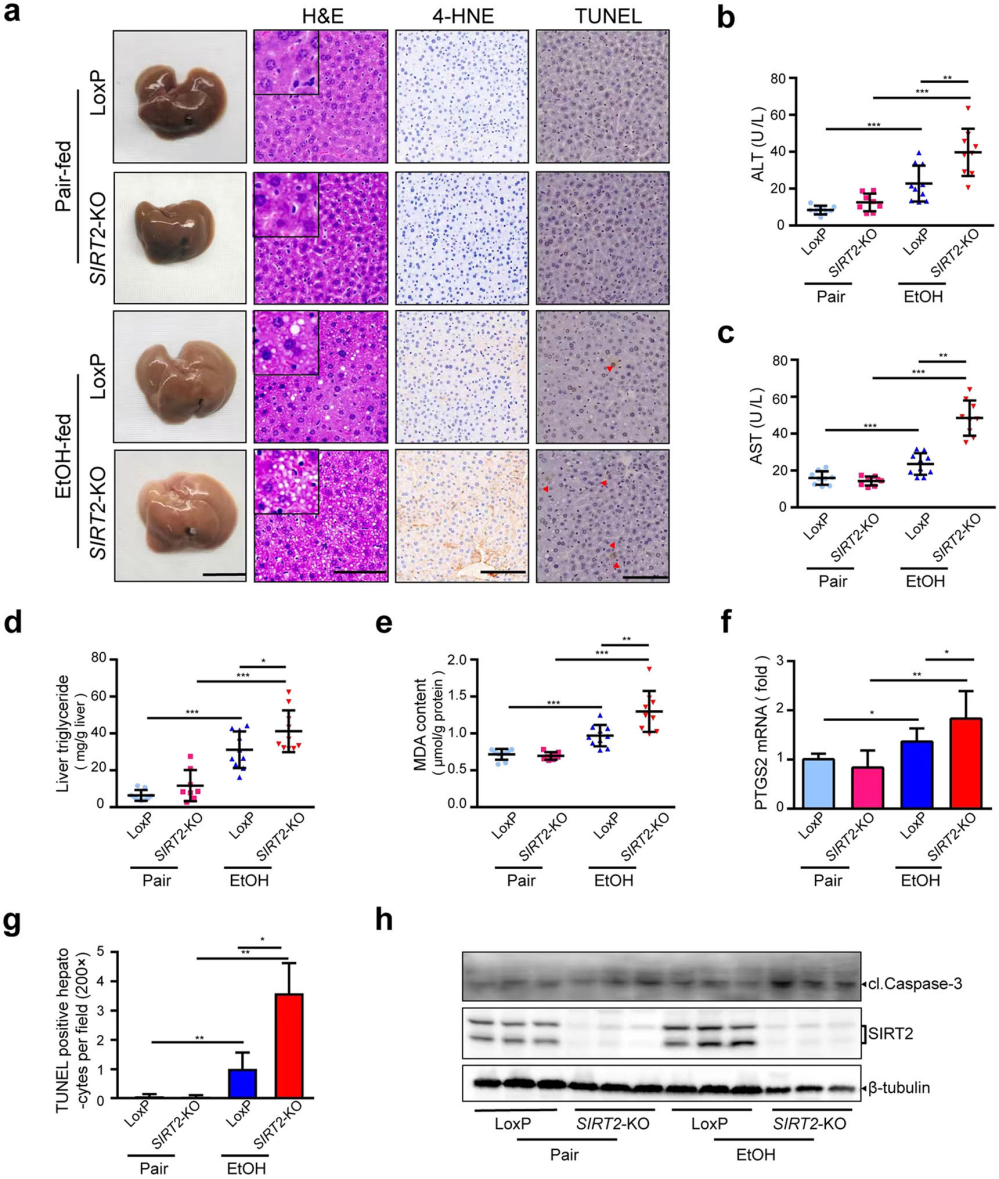
Liver-specific *SIRT2KO* sensitizes mice to alcoholic liver injury. *SIRT2*^*f/f*^*Alb*-Cre^–^ (LoxP) and *SIRT2*^*f/f*^*Alb*-Cre^+^ (*SIRT2*-KO) male mice were treated with pair (Pair) and ethanol diet (EtOH) according to NIAAA model (*n* = 8–10/group). **a**–**h** Liver injury, steatosis, lipid peroxidation, and cell apoptosis were assessed by images of the indicated livers (scale bar, 1 cm), mouse hepatic H&E staining (scale bar, 100 μm), IHC detection of 4-HNE and TUNEL (scale bar, 100 μm) (**a**), serum ALT (**b**) and AST (**c**), liver triglyceride (TG) (**d**), hepatic MDA content (**e**), and *PTGS2* mRNA (**f**), quantitative analysis of TUNEL-positive hepatocytes (magnification, ×200) (**g**), Western blot analysis of cl.Caspase-3 in murine liver tissues (**h**). Student’s *t*-test was used for statistical evaluation. Data are shown as means ± SD and are considered statistically significant at *P < 0.05, **P < 0.01^,^ and ***P < 0.001.

