# Supplementary material for: SIRT2-mediated deacetylation and deubiquitination of C/EBPβ prevents ethanol-induced liver injury
Source: Cell Discov. 2021 Oct 12;7:93. doi: 10.1038/s41421-021-00326-6 (PMC8511299; doi:10.1038/s41421-021-00326-6)
Supplement: Supplementary file 1 — Supplementary Data S1 [file 41421_2021_326_MOESM1_ESM.pdf]

Supplementary Data S1

Supplementary materials and methods

Human subjects

The study of human subjects is a retrospective case-control study conducted in the affiliated hospitals of Youjiang Medical University for Nationalities. Cases with ALD were recruited in the affiliated hospitals of Youjiang Medical University for Nationalities from January 2006 to December 2015. In this study, ALD cases were defined as the cases both with the history alcoholic drinking and with biopsy-diagnosed hepatic injury, but not including those liver injury cases caused by known non-alcoholic factors, such as hepatitis virus, drugs, and so on. The inclusion criterion cases are as follows: (1) cases with the alcoholic-drinking history, including alcohol use disorder[1]; (2) cases with the evidence of liver injury from histopathological biopsy, consisting of fatty degeneration, inflammatory change, fibrosis hyperplasia, and cirrhosis; (3) cases with available biopsy samples and clinic-pathological data for further analyses; and (4) cases understanding the objective of the study and providing informed consent. The exclusion criteria consisted of: (1) cases featuring liver injury caused by known non-alcoholic factors, such as hepatic virus, drugs, immune factors, and so on; (2) cases rejected, dropped out, or lost information; and (3) cases featuring tumors or receiving chemotherapy or radiotherapy treatment before liver biopsy because of tumors or other diseases.

According to the above inclusive and exclusive criteria, a total of 102 patients with

ALD were included for the present study. To investigate the difference of SIRT2 expression between hepatic tissues with ALD and normal liver tissues, a total of 12 paraffin-embedded hepatic tissues, which were from death patients without any evidence of liver diseases, were selected as controls from the same hospitals at the same period. Before samples collected, we obtained IRB approval. All demographic and clinicopathological information for all subjects, including gender, age, race, drinking and smoking information, and hepatic function, were obtained through the patients' medical records. This study was approved by the ethic committees of the participating hospitals and was carried out in accordance with the approved guidelines.

Histology and immunohistochemistry

After routine formalin fixed, paraffin embedded (FFPE) specimen processing, liver sections (3-4μm) were stained with hematoxylin and eosin (H&E) for histological evaluation of liver injury in patients and mice. 4-hydroxynonenal (4-HNE) staining (dilution 1:2000 for anti-4-HNE antibody) was performed for detecting lipid peroxidation. F4/80 and lymphocyte antigen 6 complex locus G6D (Ly6g) staining were performed for detecting infiltration of macrophages and neutrophils. The protein expression levels of SIRT2, C/EBPβ, LCN2, and cleaved Caspase 3 protein in the liver tissue slides of patients with AH were tested using immunohistochemistry (IHC) according to the standard procedure (protocol 40441a, Maixin Biotechnology, Inc., Fuzhou, China). The corresponding primary antibodies (dilution 1:250 for anti-SIRT2

and anti-LCN antibody, 1:300 for anti-C/EBPβ and anti-Caspase-3 antibody) and HRP-conjugated secondary antibody (catalog # KIT-9705) were obtained from Abcam Biotechnology, Inc. and Maixin Biotechnology, Inc., respectively. The quality control for IHC was administered with controls. The evaluation of IHC staining was accomplished by two independent pathologists according to the immunoreactive score (IRS) system [2]. In this study, the expression levels of SIRT2 and C/EBPβ were divided into two classifications: low (IRS value ≤ 5) and high (IRS value > 5).

Disease grade of ALD

In this study, the severity of disease was elucidated using two score models: the alcoholic hepatitis histologic score (AHHS) model[3] and liver injury score (LIS) model. AHHS model, an additive score model of the fibrosis stage, bilirubinostasis, neutrophilic granulocyte infiltration and megamitochondria, can identify the severity of AH (consisting of mild, intermediate, and severe grade) and predicate the prognosis of active ALDs. For LIS model, liver fibrosis and necrosis status for patients with ALD were evaluated by two independent pathologists. Liver fibrosis status was divided into four degrees: no fibrosis as 0-degree, fibrous portal expansion as 1-degree, bridging fibrosis (portal-portal or portal-central linkage) as 2-degree, and cirrhosis as 3-degree; whereas liver necrotic status was grouped into four degrees: no necrosis as 0-degree, spotty necrosis as 1-degree, piecemeal necrosis as 2-degree, and lobular necrosis as 3-degree. Liver proliferation status was divided into two degrees: not having hepatic proliferation as 0-degree, hepatic proliferation as 1-degree. AHHS

and LIS analyses for patients with ALD were evaluated by two independent pathologists.

Alcohol model

Mice were housed in temperature-controlled rooms with alternating 12-hour periods of light and dark. Our study used 8- to 14-week-old male mice. Age-matched mice were used in the same experiment. The National Institute on Alcohol Abuse and Alcoholism (NIAAA) model was employed as previously described [4]. All mice were firstly fed with control liquid diet for 5 days. Ethanol groups (EtOH) were then fed with a liquid diet containing 5% v/w ethanol for 10 days, whereas pair groups (Pair) were continued to be fed with control liquid diet for 10 days. The isocaloric food intake between EtOH and Pair should be provided during the ethanol feeding period. At the 11th day, EtOH was given a single oral gavage of ethanol (5 g/kg body weight, 31.25% ethanol), whereas Pair was given an isocaloric gavage of dextrin maltose. Mice were sacrificed 9 hours later. All procedures were performed in accordance with the approved guidelines by the Institutional Animal Care and Use Committee at Shanghai Jiaotong University of Medicine.

Adeno-associated Virus 8 (AAV8)-mediated gene expression

An AAV8 delivery system was used to specifically overexpress murine SIRT2, mutated SIRT2 (H187A), LCN2, C/EBPβ or mutated C/EBPβ (K102R and K211R) in mouse livers. The open reading frame encoding those genes, without a stop codon, was cloned into an AAV8 package vector pAAV-TBG-T2A-luciferase [5]. The mice

were injected 2×10^{11} viral particles of AAV8 containing either target gene or scrambled vector via the tail vein 9 days before NIAAA model construction. The target gene expression was monitored by Bioluminescence imaging (BLI) through intraperitoneal injection of D-luciferin (150μg/g BW).

Serum biochemistry and liver triglyceride (TG) and malondialdehyde (MDA) analysis

Serum alanine transaminase (ALT), aspartate aminotransferase (AST) and liver triglyceride (TG) concentrations were measured with ALT, AST and TG assay kits (Jiancheng Bioengineering Institute, Nanjing, China). Liver concentration of malondialdehyde (MDA), one of the end products of lipid peroxidation, was measured with a lipid peroxidation MDA Assay Kit (Beyotime Biotechnology, Shanghai, China). All of these analyses were performed with a Spark microplate reader (Tecan, Swit).

Apoptosis assay

The apoptotic cells in liver sections were detected using a commercial terminal deoxynucleotidyl transferase-mediated dUTP nick end labeling (TUNEL) staining according to the manufacturer's instructions. The number of TUNEL-positive cells was counted in ten randomly selected fields around the centrilobular areas (×200) of similar size, per slide, in a double-blinded manner and calculated, as the total number of TUNEL-positive cells/ten fields.

RNA extraction and real-time quantitative PCR (qRT-PCR)

Total RNA was extracted from tissues and cells using TRIzol (Invitrogen) according to the manufactuer's instructions. The purified RNA (1 μg) was quantified and reverse-transcribed using AMV Reverse Transcriptase XL (Takara, Mountainc View, CA). Details of qRT-PCR have previously been described [6,7]. Real-time quantitative PCR was performed using SYBR Green Master Mix (Applied Biosystems, Foster City, CA). The expression levels of the mRNA transcripts were calculated relative to the expression of RPL13A by using the formula $2^{-\Delta\Delta Ct}$. The primer sequences are listed in Supplementary CTAT table.

Western blot

Whole cell lysates prepared from mouse livers and cells were described previously[7-9].The nuclear and cytoplasmic extracts from cells were obtained using an NE-PER™ Nuclear Cytoplasmic Extraction Reagent kit (Thermo Fisher Scientific) according to the manufacturer's instructions. Equal amounts of proteins were separated by SDS-PAGE and blotted to nitrocellulose membranes (Bio-rad, Richmond, CA). Target proteins were detected by immunoblotting with specific primary antibodies and horseradish peroxidase (HRP)-labeled secondary antibody. Immobilon Western Chemiluminescent HRP substrate kit (Millipore) was used for detection. The protein levels were quantified by gray scanning.

RNA-seq

RNA from liver tissus (20mg) was extracted and quantified using a Nano Drop (Thermo Fish Scientific, MA, USA). Oligo(dT)-attached magnetic beads were used to

purify mRNA. Purified mRNA was fragmented into small pieces with fragment buffer at appropriate temperature. Then First-strand cDNA was generated using random hexamer-primed reverse transcription, followed by a second-strand cDNA synthesis. afterwards, A-Tailing Mix and RNA Index Adapters were added by incubating to end repair. The cDNA fragments obtained from previous step were amplified by PCR, and products were purified by Ampure XP Beads, then dissolved in EB solution. The product was validated on the Agilent Technologies 2100 bioanalyzer for quality control. The double stranded PCR products from previous step were heat-denatured and circularized by the splint oligo sequence to get the final library. The single strand circular DNA (ssCir DNA) was formatted as the final library. The final library was amplified with phi29 to make DNA nanoball (DNB) which had more than 300 copies of one molecular, DNBs were loaded into the patterned nanoarray and single end 50-base reads were generated on BGIseq500 platform (BGI-Shenzhen, China).

<u>Luciferase reporter assay</u>

Luciferase reporter assays were performed as previously described [10]. Dual luciferase reporter gene vector containing 3-kb LCN2 promoter or LCN2 T-150 promoter truncation (deletion -150bp~-1bp from 3-kb LCN2 promoter, -151bp~-3000bp) was constructed. Cells were seeded in 12-well plates overnight prior to transfection. Human embryonic kidney 293T (HEK293T) cells were co-transfected with 500ng of LCN2 promoter luciferase reporter, 1μg of empty vector or C/EBPβ expression plasmid, and 20ng of Renilla reporter. Forty-eight hours after

transfection, the cells were harvested in lysis buffer. The Dual Light Reporter System (Applied Biosystems) was used to detect reporter expression, followed by calculation of the luciferase to renilla ratio.

Chromatin Immunoprecipitation

UCSC Epigenome Browser tracks of the C/EBPβ ChIP-Seq signal -3 kb before the transcription start site (TSS) of LCN2 in mouse liver from Cistrome DB Toolkit (http://cistrome.org/db/#/) [11]. C/EBPβ binging sites were predicted on the promoters of LCN2 by the analysis of PROMO (http://acgt.cs.tau.ac.il/promo/). ChIP analysis was performed using the Millipore ChIP Assay Kit (Millipore, MA, USA) according to the manufacturer's instructions. Details of ChIP have previously been described [6]. In brief, ChIP was performed with 5×10^6 cells per reaction. Cells were crosslinked with formaldehyde and sonicated. Corresponding IgG was used as controls. ChIP DNA was purified using QIAquick Spin Columns (Qiagen). The purified ChIP DNA was quantified by qRT-PCR. The primers used are listed in the Supplementary CTAT table.

Confocal microscopy-immunofluorescence

AML12 cells were seeded on coverslipsin 24-well plates overnight. Treated by alcohol for 48 hours, cells were fixed in 4% paraformaldehyde at room temperature for 10min and permeabilized in methanol at 4 ℃ for 15min. Then, coverslips were rinsed in PBS for three times and blocked in 1% bovine serum albumin (BSA) at room temperature for 1 h. Target protein expressions and locations were detected by

incubating with primary antibodies overnight at 4℃ in a humid chamber. After washing five times, secondary antibodies (Texas-Red or FITC tagged) were applied in a 1:200 dilution in staining buffer for 1h at 37 ℃ in a humid chamber in the dark. After washing, coverslips were mounted with Vectorshield with 4′,6-diamidino-2-phenylindole (DAPI; Vector Laboratories, CA), and analyzed on a Nikon Eclipse TI Laser Scanning Microscope or Leica TCS SP8.

<u>Immunoprecipitation</u>

Immunoprecipitation were conducted as previously described [8,12]. Cells and murine liver tissues were harvested, lysed, and briefly sonicated at 4 ℃. The supernatants (whole-cell lysates) were collected and incubated with anti-FLAG M2 beads (Sigma) or anti-C/EBPβ antibody at 4℃ overnight. The precipitates were washed three times with immunoprecipitation buffer (50 mM Tris-HCl, PH 7.6, 150 mM NaCl, 1mM EDTA, 1% NP-40, 1 mM PMSF, and 1x protease inhibitor cocktail (Calbiochem)), boiled in sample buffer and subjected to Western blot analysis.

<u>In vivo ubiquitination assay</u>

In vivo ubiquitination assay were conducted as previously described [8]. In brief, AML12 cells and primary heptocytes isolated from LoxP mice and *SIRT2*-KO mice were treated with 10 μM of the proteasome inhibitor MG132 (Sigma) for 6h before lysis and sonication. The supernatants were collected and subjected to immunoprecipitation with anti-FLAG M2 beads or anti-C/EBPβ antibody, followed by Western blot analysis to visualize polyubiquitylated protein bands.

Isolation of primary hepatocyte

Primary hepatocytes were isolated, as previously reported[13]. Briefly, mice were anesthetized, and the livers were perfused *in situ* through portal vein puncture and flowed firstly with the perfusion buffer (0.075% sodium Bicarbonate solution in Hanks' Balanced Salt Solution (HBSS, no Ca^{2+}/Mg^{2+}) buffer with 0.13% EDTA) at 37℃ at a rate of 2.8-3.0 ml/min, followed by the digestion buffer (collagenase type II in HBSS (with Ca^{2+}/Mg^{2+}) containing 0.075% sodium Bicarbonate solution and 5mM $CaCl_2$) at 37 ℃ at a rate of 1.4 - 1.5 ml/min. After digestion, liver tissues were cut into pieces and suspended with DMEM. The suspension containing dissociated cells were collected and filtered through a 100 μm cell strainer (BD Biosciences, San Jose, CA) followed by centrifuge at 150 g for 3 min at 4℃. After this step, viable hepatocyte will be at the bottom of the tubes and collected for the subsequent analysis.

Nano-LC−ESI-MS/MS analysis

Nano-LC−MS/MS with electrospray ionization was used to identify interacting proteins as previously described [10]. In brief, AML12 cells stably transfected SIRT2-targeting shRNA vector (AML12 shSIRT2 and AML12 shNC) were transfected with Flag-tagged C/EBPβ expression plasmid. Forty-eight hours after transfection, the cells were lysed, and briefly sonicated. Mixed protein lysates were subjected to immunoprecipitation with anti-FLAG M2 beads. Immunoprecipitation samples were separated by SDS-polyacrylamide gel electrophoresis, and visualized

with colloidal Coomassie blue. The target lane from gels was prepared for analysis by LC-MS/MS. The MS spectrum was acquired using an Orbitrap Fusion LUMOS mass spectrometer (Thermo Fisher Scientific) connected to an Easy-nLC 1200 via an Easy Spray (Thermo Fisher Scientific).

Cell lines and cell culture

HEK293T cells and mouse liver AML12 cell lines were purchased from the American Type Culture Center and subcultured precisely per American Type Culture Center protocol. 293T cells were maintained in Dulbecco's modifed Eagle's medium (DMEM) supplemented with 10% fetal bovine serum (FBS). AML12 cells were maintained in DMEM:F12 Medium supplemented with 10% FBS, 10 μg/ml insulin, 5.5 μg/ml transferring, 5 ng/ml selenium and 40 ng/ml dexamethasone. No signs of mycoplasma contamination were found for all cell lines.

Plasmids, siRNA, shRNA, and antibodies

Information on the plasmids, siRNA, shRNA and antibodies is provided in the Supplementary Tables S3-4.

References

1 Crabb, D. W., Im, G. Y., Szabo, G., Mellinger, J. L. & Lucey, M. R. Diagnosis and Treatment of Alcohol-Associated Liver Diseases: 2019 Practice Guidance From the American Association for the Study of Liver Diseases. *Hepatology* **71**, 306-333 (2020).

2 Friedrichs, K., Gluba, S., Eidtmann, H. & Jonat, W. Overexpression of p53 and prognosis in breast cancer. *Cancer* **72**, 3641-3647 (1993).

3 Altamirano, J. *et al.* A histologic scoring system for prognosis of patients with alcoholic hepatitis. *Gastroenterology* **146**, 1231-1239 e1231-1236 (2014).

4 Bertola, A., Mathews, S., Ki, S. H., Wang, H. & Gao, B. Mouse model of chronic and binge ethanol feeding (the NIAAA model). *Nat Protoc* **8**, 627-637 (2013).

5 Fan, W. *et al.* ECM1 Prevents Activation of Transforming Growth Factor beta, Hepatic Stellate Cells, and Fibrogenesis in Mice. *Gastroenterology* **157** (2019).

6 Shen, S. M. *et al.* Nuclear PTEN safeguards pre-mRNA splicing to link Golgi apparatus for its tumor suppressive role. *Nat Commun* **9**, 2392 (2018).

7 He, M. *et al.* HIF-1alpha downregulates miR-17/20a directly targeting p21 and STAT3: a role in myeloid leukemic cell differentiation. *Cell Death Differ* **20** (2013).

8 He, M. *et al.* An Acetylation Switch of the NLRP3 Inflammasome Regulates Aging-Associated Chronic Inflammation and Insulin Resistance. *Cell Metab* **31**, 580-591 e585 (2020).

9 Shin, J. *et al.* SIRT7 represses Myc activity to suppress ER stress and prevent fatty liver disease. *Cell Rep* **5**, 654-665 (2013).

10 Ge, M. K. *et al.* FBXO22 degrades nuclear PTEN to promote tumorigenesis. *Nat Commun* **11**, 1720 (2020).

11 Zheng, R. *et al.* Cistrome Data Browser: expanded datasets and new tools for gene regulatory analysis. *Nucleic Acids Res* **47**, D729-D735 (2019).

12 Jiang, W. *et al.* Acetylation regulates gluconeogenesis by promoting PEPCK1 degradation via recruiting the UBR5 ubiquitin ligase. *Mol Cell* **43**, 33-44 (2011).

13 Zhao, X. Y. *et al.* Long noncoding RNA licensing of obesity-linked hepatic lipogenesis and NAFLD pathogenesis. *Nat Commun* **9**, 2986 (2018).
