# Supplementary material for: SIRT2-mediated deacetylation and deubiquitination of C/EBPβ prevents ethanol-induced liver injury
Source: Cell Discov. 2021 Oct 12;7:93. doi: 10.1038/s41421-021-00326-6 (PMC8511299; doi:10.1038/s41421-021-00326-6)
Supplement: Supplementary file 2 — Supplementary information [file 41421_2021_326_MOESM2_ESM.pdf]

Supplementary figures

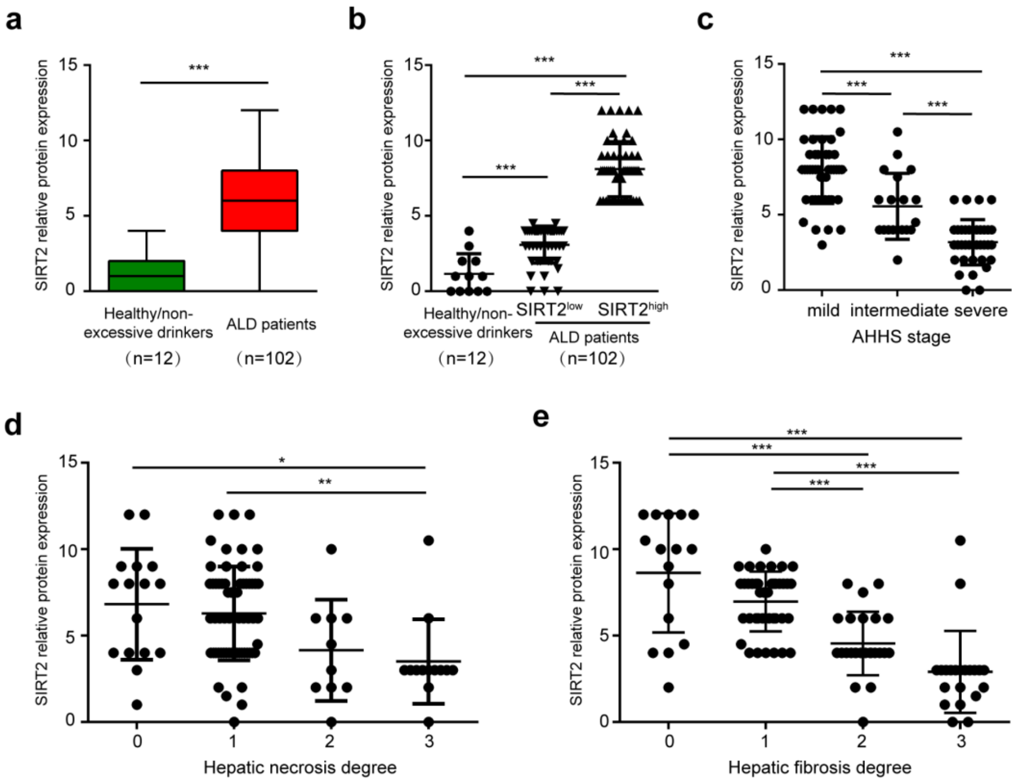

Supplementary Fig. S1, related to Fig. 1. SIRT2 protein expression was reversely correlated with hepatic necrosis and fibrosis in ALD patients. a-b SIRT2 relative protein expression in the liver samples of the normal controls and ALD patients (containing SIRT2^{low} and SIRT2^{high} patients). **c-e** SIRT2 relative protein expression with AHHS stage (**c**), different degree of hepatic necrosis (d) and hepatic fibrosis (e). Statistical significance was calculated by Student's *t* test for two-sample comparisons and one-way ANOVA was used for multiple comparisons. Data were shown as mean ± SD and were considered statistically significant at $^{*}P< 0.05$, $^{**}P< 0.01$, and $^{***}P<0.001$.

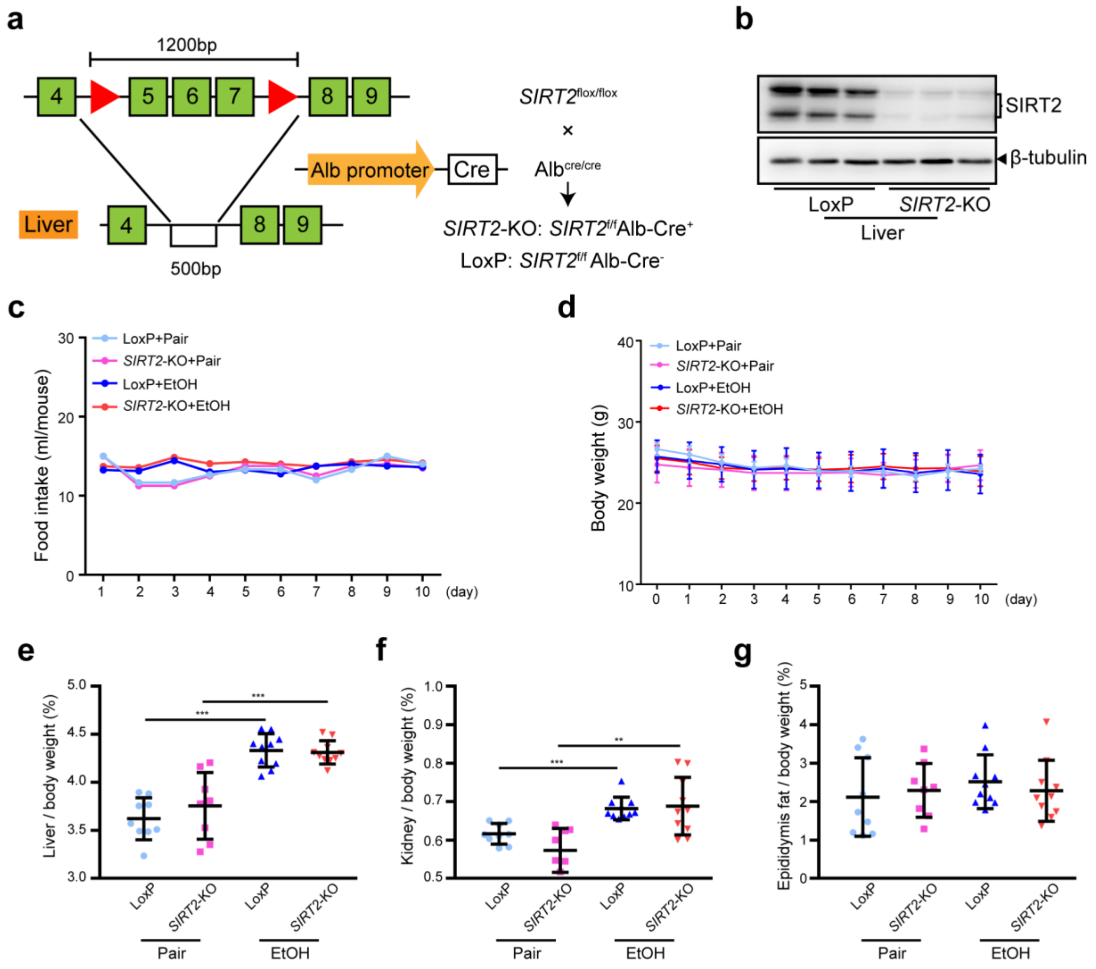

Supplementary Fig. S2, related to Fig. 2. Liver-specific SIRT2 deficiency has no effect on food intake, body weight and organ to body weight ratios. a Scheme for the generation of liver-specific SIRT2 knockout mice (*SIRT2*-KO). **b** western blot confirmed deficiency of SIRT2 in the livers of *SIRT2*-KO mice. LoxP and *SIRT2*-KO male mice were treated with NIAAA model (n=8-10/group). **c-f** Food intake (**c**) and body weight (**d**) were monitored during 10 days with a liquid diet containing 5% v/w ethanol or pair fed. Liver-specific SIRT2 deficiency has no effect on alcohol-induced increase of liver/body weight ratios (**e**), kidney/body weight ratios (**f**). **g** Alcohol does not affect epididymal fat/body weight ratios. Data were shown as mean ± SD and were considered statistically significant at $^{**}P < 0.01$, and $^{***}P < 0.001$.

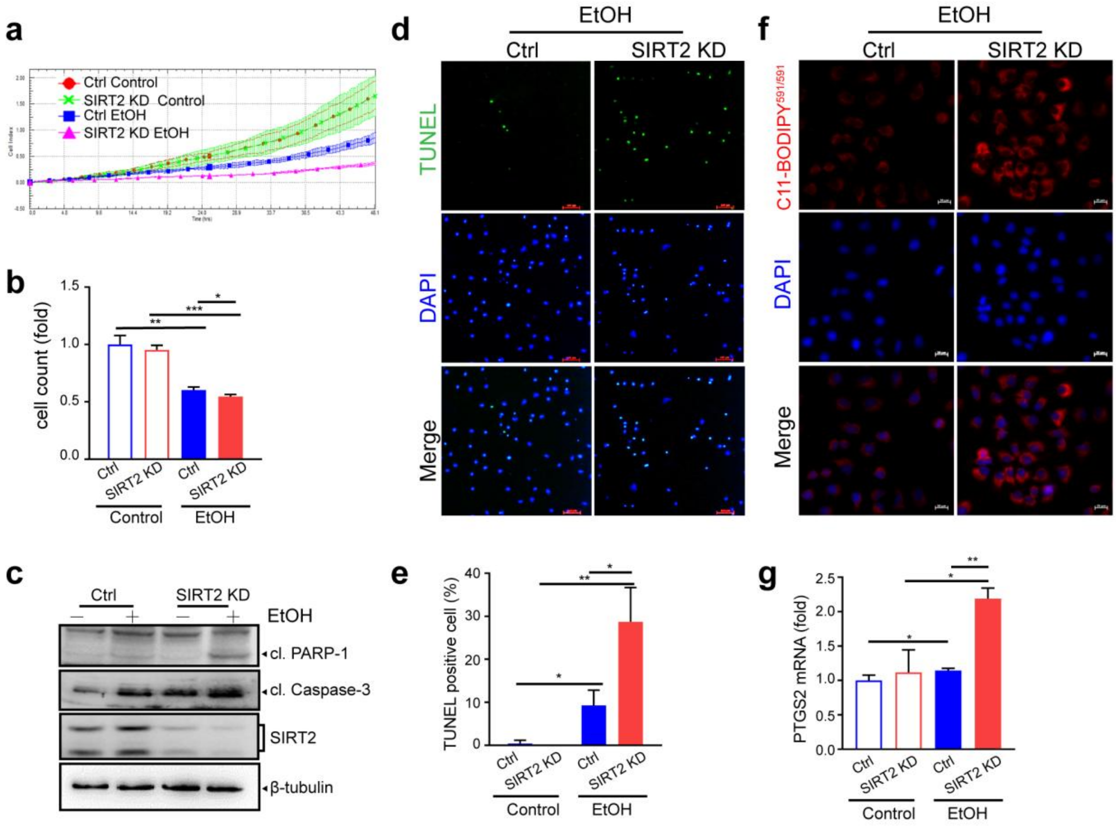

Supplementary Fig. S3. SIRT2 knockdown leads to more apoptosis and lipid peroxidation in AML12 hepatocytes under ethanol exposure. SIRT2 knockdown (SIRT2 KD) and control (Ctrl) AML12 cells were treated with EtOH (250 μM) for 48 hours. **a** Dynamic monitoring of cell proliferation using the xCELLigence system. **b** Cell count analysis. **c** western blot analysis of Caspase-3 and PARP-1 cleavage. **d** Immunofluorescence analysis of TUNEL-positive cells (scale bar, 100 μm). **e** Quantitative analysis of TUNEL-positive hepatocytes. **f** Immunofluorescence analysis of lipid peroxidation through C11-BODIPY$^{581/591}$ staining. **g** *PTGS2* mRNA analysis by qRT-PCR. Student's *t* test was used for statistical evaluation. Data were shown as mean ± SD and were considered statistically significant at $^{*}P < 0.05$, $^{**}P < 0.01$, and $^{***}P<0.001$.

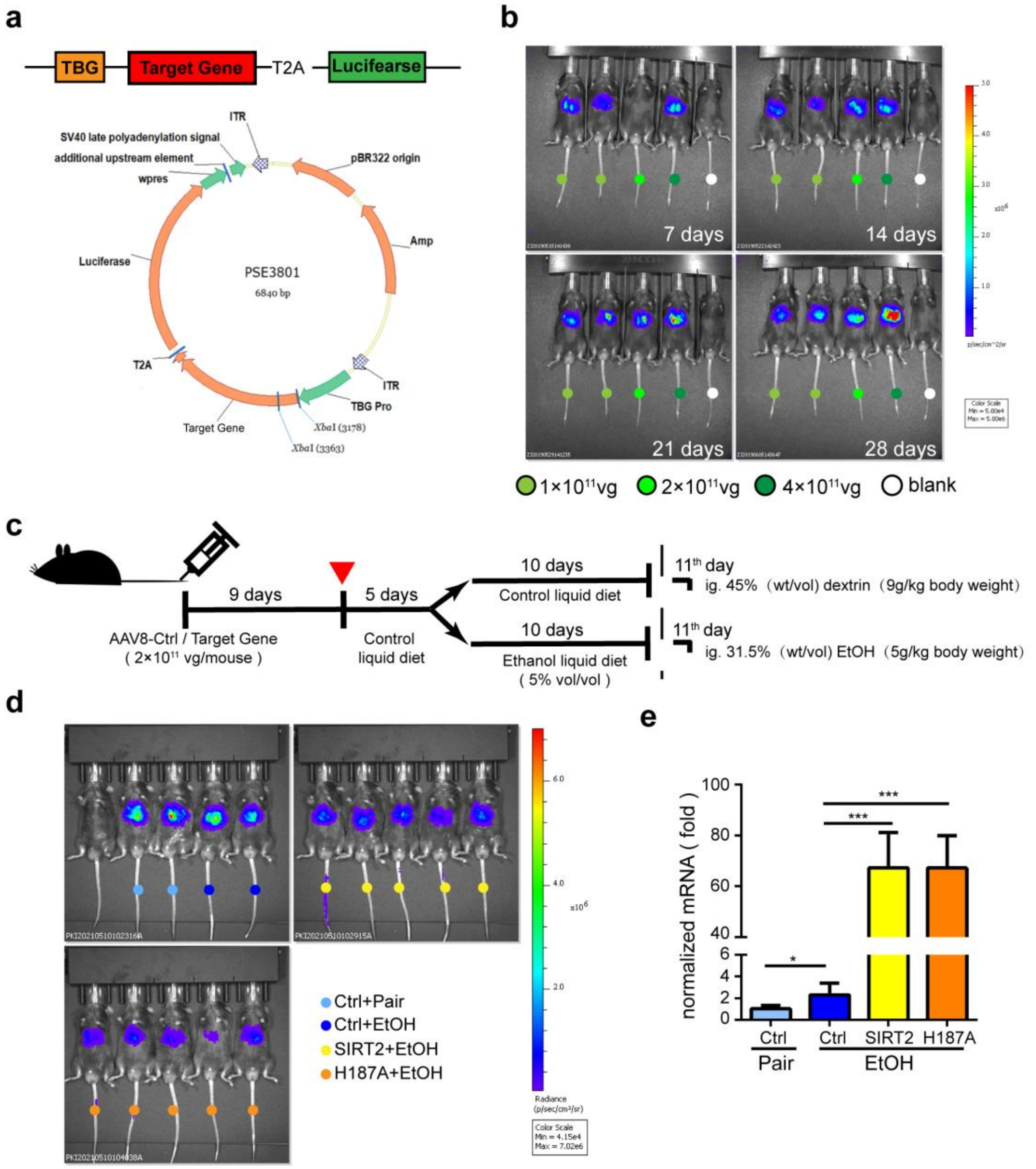

Supplementary Fig. S4, related to Fig. 3. Generation of hepatocyte-specific SIRT2 overexpression mouse model. a Diagram for the hepatocyte-specific serotype 8 recombinant adeno-associated virus (AAV8) delivery system of target gene overexpression. **b** *In vivo* bioluminescence images (BLI) pictured weekly from WT mice tail-injected with 1×10^{11}, 2×10^{11} or 4×10^{11} AAV8-TBG-luciferase (Ctrl) viral particles. **c** Schematic representation of AAV8-target gene treatment model. **d** BLI of AAV8 liver-specific expression of Ctrl, AAV8-TBG-SIRT2 (SIRT2) or AAV8-TBG-SIRT2-H187A (H187A) following NIAAA model at 9th day after tail-injection. **e** Hepatic normalized SIRT2 mRNA expression analysis by qRT-PCR.

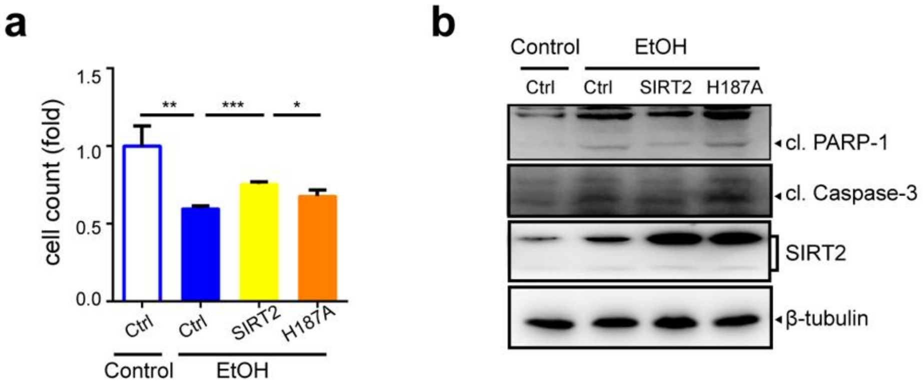

Supplementary Fig. S5. SIRT2 overexpression leads to less apoptosis in AML12 hepatocytes under ethanol exposure. AML12 cells with SIRT2 overexpression (SIRT2) or SIRT2-H187A overexpression (H187A) or control (Ctrl) was treated with EtOH (250 μM) for 48 hours. **a** Cell count analysis. **b** western blot analysis of Caspase-3 and PARP-1 cleavage. Data were shown as mean ± SD and were considered statistically significant at $^{*}P < 0.05$, $^{**}P < 0.01$, and $^{***}P < 0.001$.

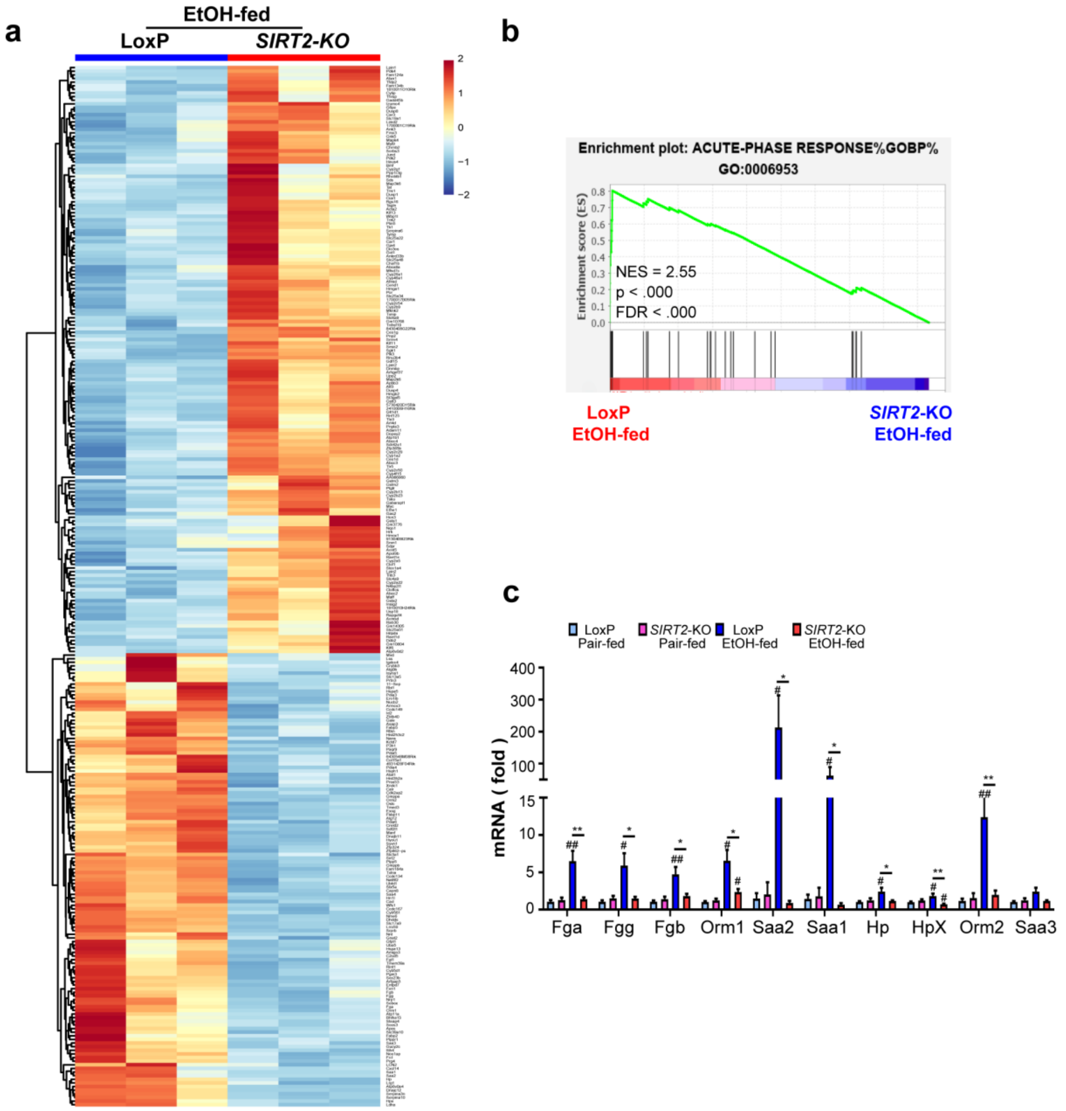

Supplementary Fig. S6, related to Fig. 4. Liver-specific SIRT2 deficiency inhibits ethanol-induced up-regulation of acute phase proteins (APPs). a Heatmap of differentially expressed genes identified by RNA-seq using liver tissues from EtOH-fed LoxP and *SIRT2*-KO mice. **b** Overview of gene set enrichment analysis (GSEA) used to identify the functional roles of SIRT2 in alcoholic liver injury. The top ranked pathway affected by *SIRT2*-KO was acute-phase response. **c** The inhibition of mRNAs of APPs found by RNA-seq in EtOH-fed *SIRT2*-KO mice was verified by qRT-PCR analysis. Data were shown as mean ± SD and were considered statistically significant at $^{*}P < 0.05$, $^{**}P < 0.01$, and $^{***}P<0.001$; $^{\#}P<0.05$ or $^{\#\#}P<0.01$ compared with Pair-fed LoxP group.

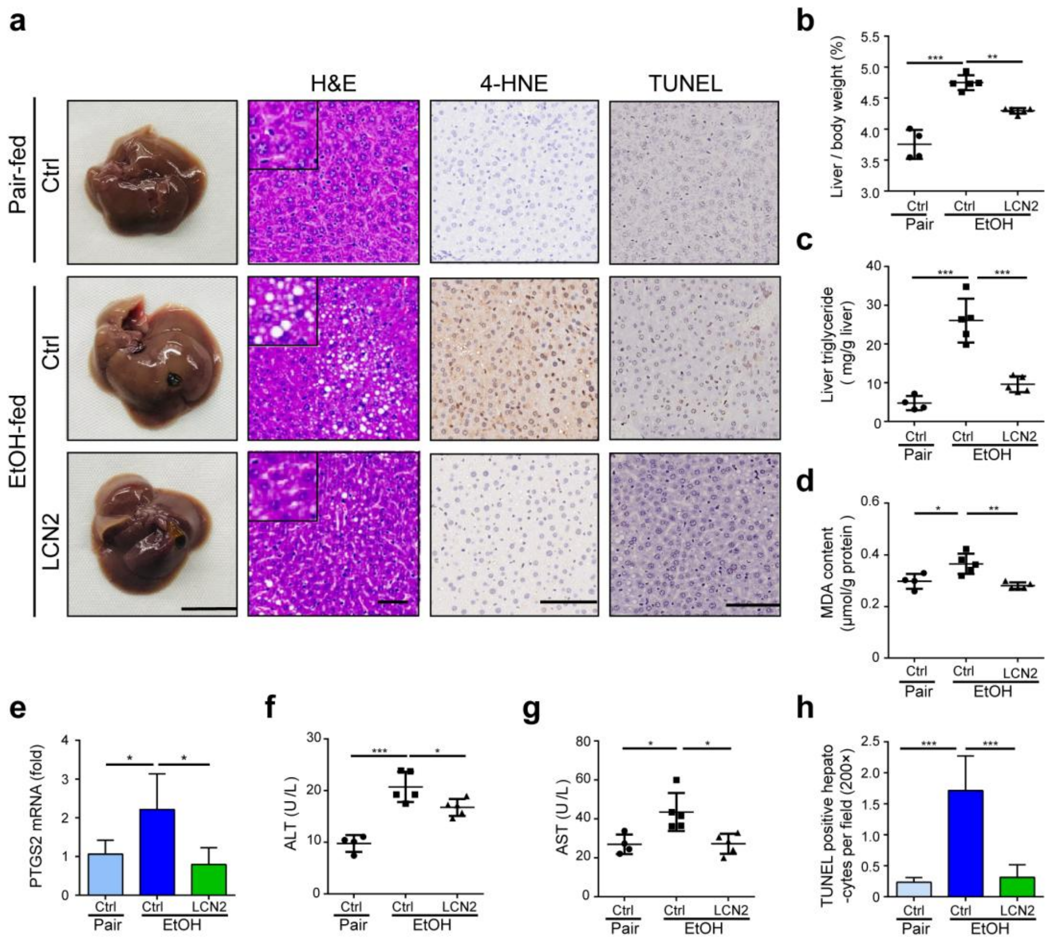

Supplementary Fig. S7, related to Fig. 4. Hepatic LCN2 reverses alcoholic liver injury in mice. LoxP Mice (12 weeks) tail-injected with AAV8-Ctrl (Ctrl) or AAV8-LCN2 (LCN2) were treated with NIAAA model (n=4~5/group). The effects of LCN2 on steatosis, lipid peroxidation, and hepatocyte apoptosis were assessed by **a** images of the indicated livers (scale bar, 1 cm), hepatic H&E staining (scale bar, 50 μm), IHC detection of 4-HNE and TUNEL (scale bar, 100 μm), **b** liver/body weight ratios, **c** liver TG, **d** hepatic MDA content and **e** *PTGS2* mRNA, **f** serum ALT and **g** AST, **h** quantitative analysis of TUNEL positive hepatocytes (magnification, ×200). Student's *t* test was used for statistical evaluation. Data were shown as mean ± SD and were considered statistically significant at $^{*}P< 0.05$, $^{**}P< 0.01$, and $^{***}P<0.001$.

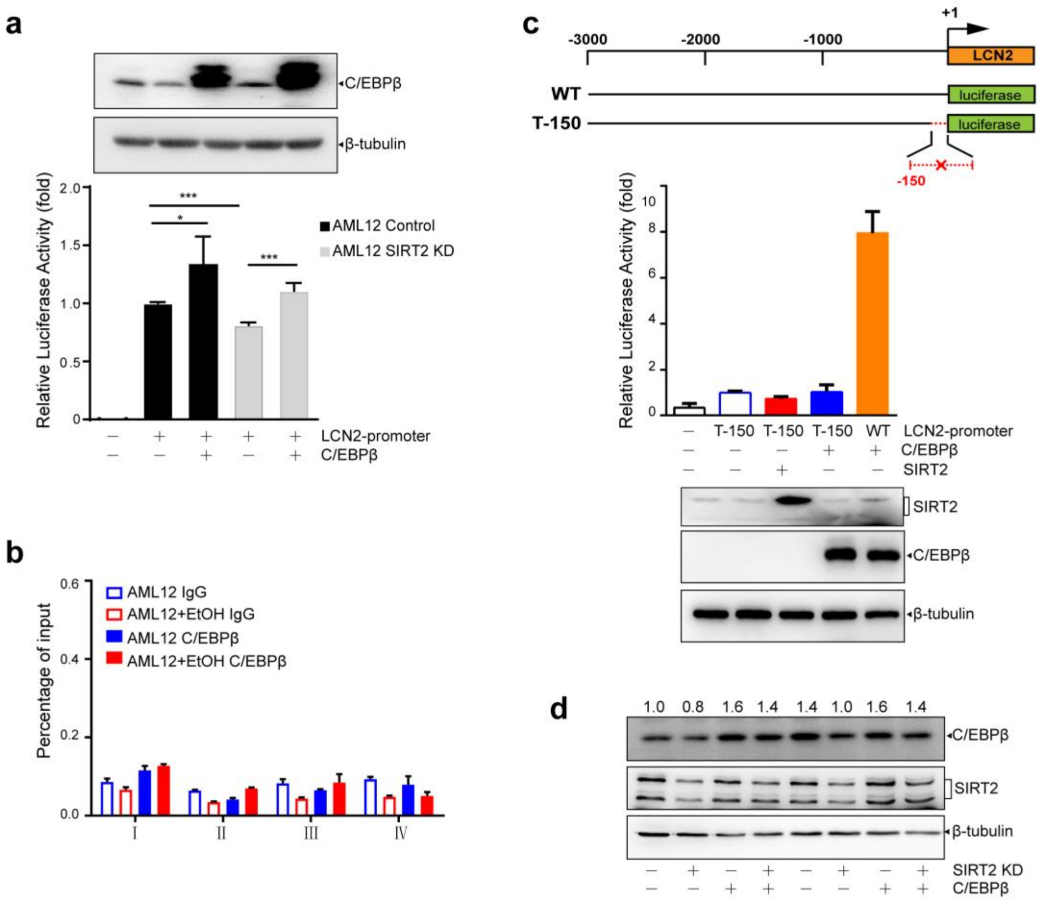

Supplementary Fig. S8, related to Fig. 5. SIRT2 up-regulates LCN2 transcription through C/EBPβ. a western blot analysis of C/EBPβ expression in the SIRT2 KD and control AML12 cells transfected with C/EBPβ plasmid (top); the luciferase activities of LCN2 promoter reporter in AML12 hepatocytes (bottom). Student's *t* test was used for statistical evaluation. Data were shown as mean ± SD and were considered statistically significant at $^{*}P< 0.05$, $^{**}P< 0.01$, and $^{***}P<0.001$. **b** ChIP analysis of C/EBPβ on the promoters (Ⅰ-IV) of LCN2 in AML12 cells treated with EtOH. **c** Schematic representation of LCN2 WT and T-150 promoter truncation (top); the luciferase activities of LCN2 promoter reporters in 293T cells transfected with SIRT2 or C/EBPβ plasmid (middle); western blot analysis of SIRT2 and C/EBPβ expression (bottom). **d** western blot analysis of C/EBPβ expression in SIRT2 KD AML12 cells transfected with C/EBPβ, related to Fig. 5g. Cells were pretreated EtOH for 48 hours.

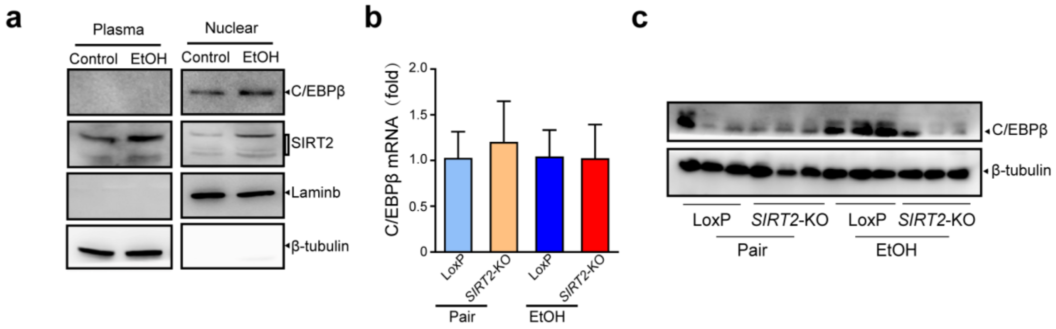

Supplementary Fig. S9, related to Fig. 6. SIRT2 up-regulates C/EBPβ at post-transcription level *in vivo* and *in vitro*. a western blot analysis of C/EBPβ and SIRT2 protein expression in the cytoplasmic and nuclear fractions of AML12 cells treated with EtOH for 48 hours. **b-c** qRT-PCR and western blot analysis of C/EBPβ expression in the liver tissues from LoxP and *SIRT2*-KO mice fed with pair or EtOH diet.

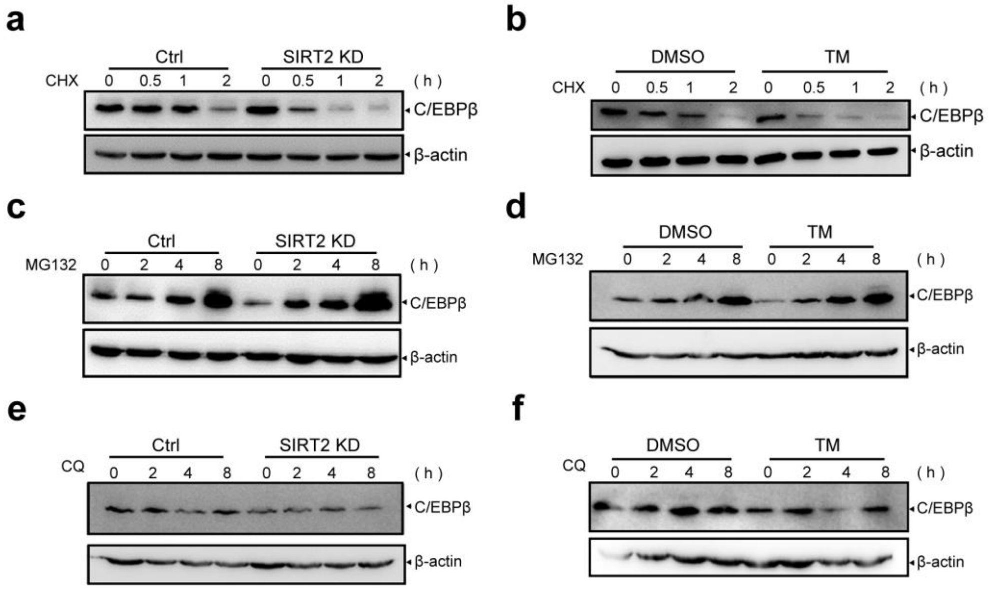

Supplementary Fig. S10, related to Fig. 6. SIRT2 stabilized C/EBPβ through inhibiting proteasome-mediated degradation. a-f ALM12 cells pretransfected with SIRT2 shRNA or SIRT2 inhibitor thiomyristoyl (TM, 1μM) were treated with 50 μg/ml cycloheximide (CHX) (**a-b**), or with 10 μM proteasome inhibitor MG132 (**c-d**), or with 50 μM lysosome inhibitor chloroquine (CQ) (**e-f**), followed by western blot of C/EBPβ protein.

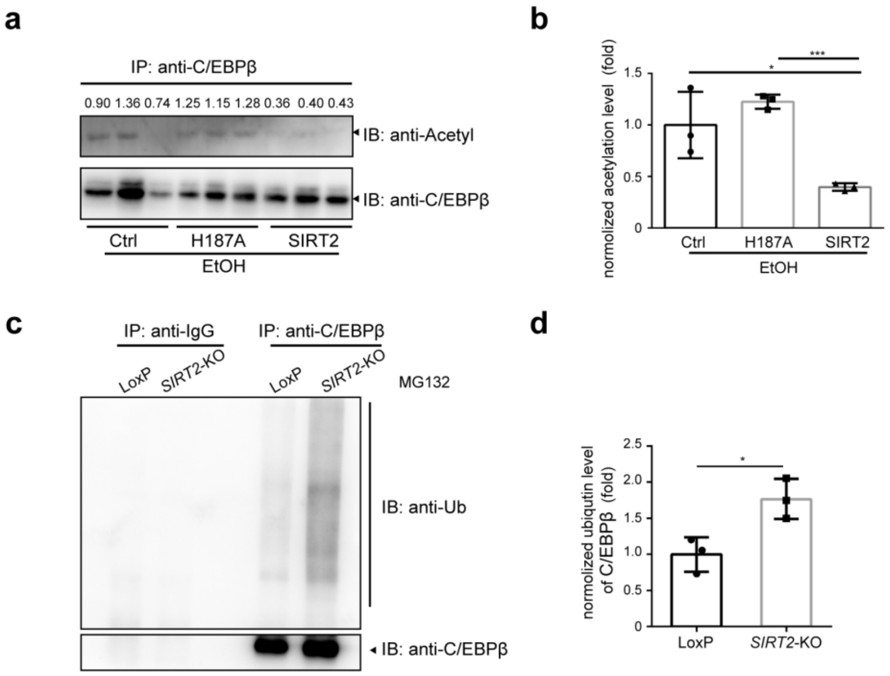

Supplementary Fig. S11, related to Fig. 6. SIRT2 deacetylates C/EBPβ and decreases C/EBPβ ubiquitination *in vivo*. a Acetylation levels of IP purified C/EBPβ in the liver tissues of the mice tail-injected with AAV8-Ctrl (Ctrl) or AAV8-SIRT2 (SIRT2) or AAV8-H187A (H187A) and treated with NIAAA model. **b** The quantitative analysis of the acetylation levels of C/EBPβ by gray scanning. **c** Primary hepatocytes isolated from LoxP and *SIRT2*-KO mice were treated with 10 μM MG132 for 6 h. The ubiquitination levels of IP purified endogenous C/EBPβ were probed by pan-ubiquitin antibody. **d** The quantitative analysis of the ubiquitination levels of C/EBPβ by gray scanning. Data were shown as mean ± SD and were considered statistically significant at $^{*}P < 0.05$ and $^{***}P < 0.001$.

a

Location	Peptide	Control (*E+06)			SIRT2 KD (*E+06)			P value
102	K(+42.01)PADYGYVSLGR	0	0	9.53	21.0	22.0	16.4	0.01
133	AAPAC(+57.02)FPPPPPAALK(+42.01) AEPGFEPADC(+57.02)KR	0	0	0	6.29	0	0	0.37
211	AAPAAC(+57.02)FAGPPAAPAK(+42.01)AK	0	0	0	8.78	6.45	1.13	0.07
220	TVDK(+42.01)LSDEYK	0	0	0	1.08	0	0	0.37
283	R.NLFK(+42.01)QLPEPLLASAGHC(+57.02)	0	0	0	0	0	0	1.00

b

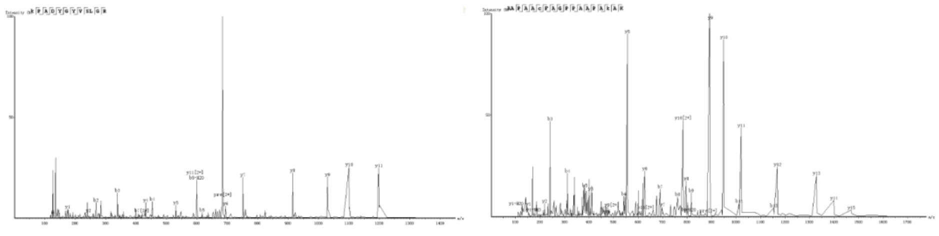

K(+42.01)PADYGYVSLGR

AAPAAC(+57.02)FAGPPAAPAK(+42.01)AK

c

Mouse	90	LFADDYGAKPSKKPA......CFAGPPAAPA - - KAKAKK
Rat	91	LFADDYGAKPSKKPS......CFAGPPAAPA - - KAKAKK
Human	121	LFSDDYGGKNCKKPA......CYAGAAPAPSQVKSKAKK
Chick	106	LFAEDYKGSGGGKKP......GYSGPPA - - - -GKNKPKK
Bovin	121	LFSDDYGGKNCKKAA......CYAGAAPAPSQVKSKAKK

Supplementary Fig. S12, related to Fig. 6. SIRT2 decreases C/EBPβ ubiquitination by deacetylating K102 and K211. a LC-MS/MS analysis for acetylated sites of Flag-C/EBPβ immunopurified from AML12 cells treated with control or SIRT2 siRNA. **b** Tandem mass spectrum of two peptides showing K102 (left) and K211 (right) of C/EBPβ deacetylated by SIRT2. **c** K102 and K211 of C/EBPβ are conserved across species. Sequence alignment of C/EBPβ from various mammalian species is shown. K102 and K211 are labeled with gray square.

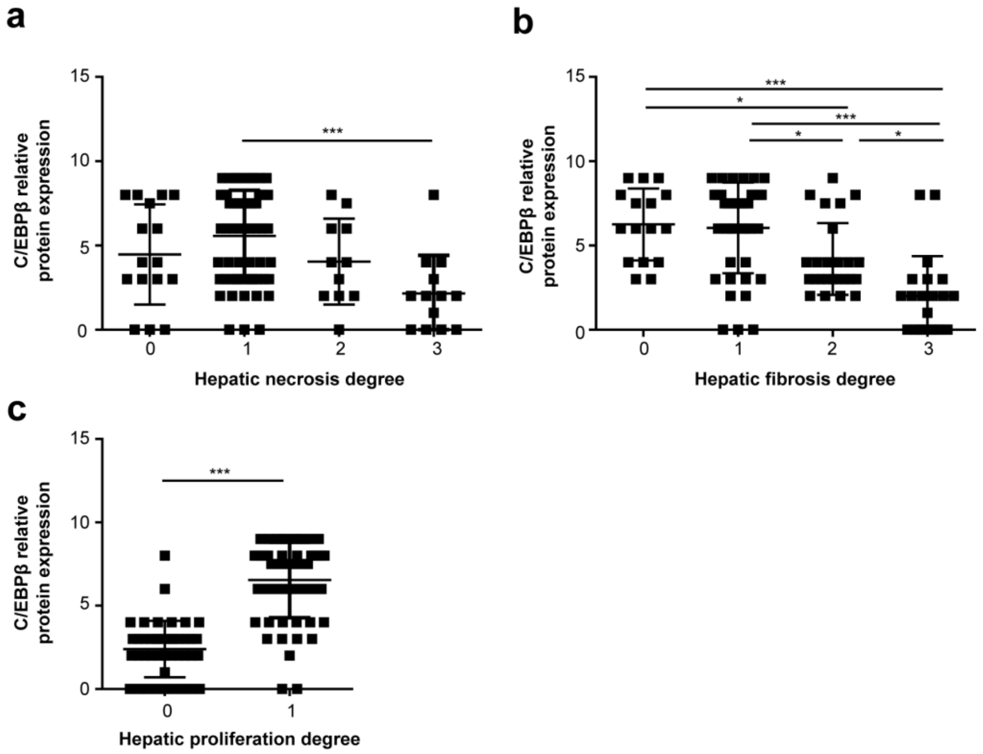

Supplementary Fig. S13, related to Fig. 8. C/EBPβ protein expression reversely correlates with hepatic necrosis and fibrosis, and positively correlates with hepatic proliferation in ALD patients. a-c C/EBPβ relative protein expression in liver tissues with different degree of hepatic necrosis (**a**), hepatic fibrosis (**b**) and hepatic proliferation (**c**). Statistical significance was calculated by Student's *t* test for two-sample comparisons and one-way ANOVA was used for multiple comparisons. Data were shown as mean ± SD and were considered statistically significant at $^{*}P<$ 0.05, $^{**}P<$ 0.01, and $^{***}P<$0.001.

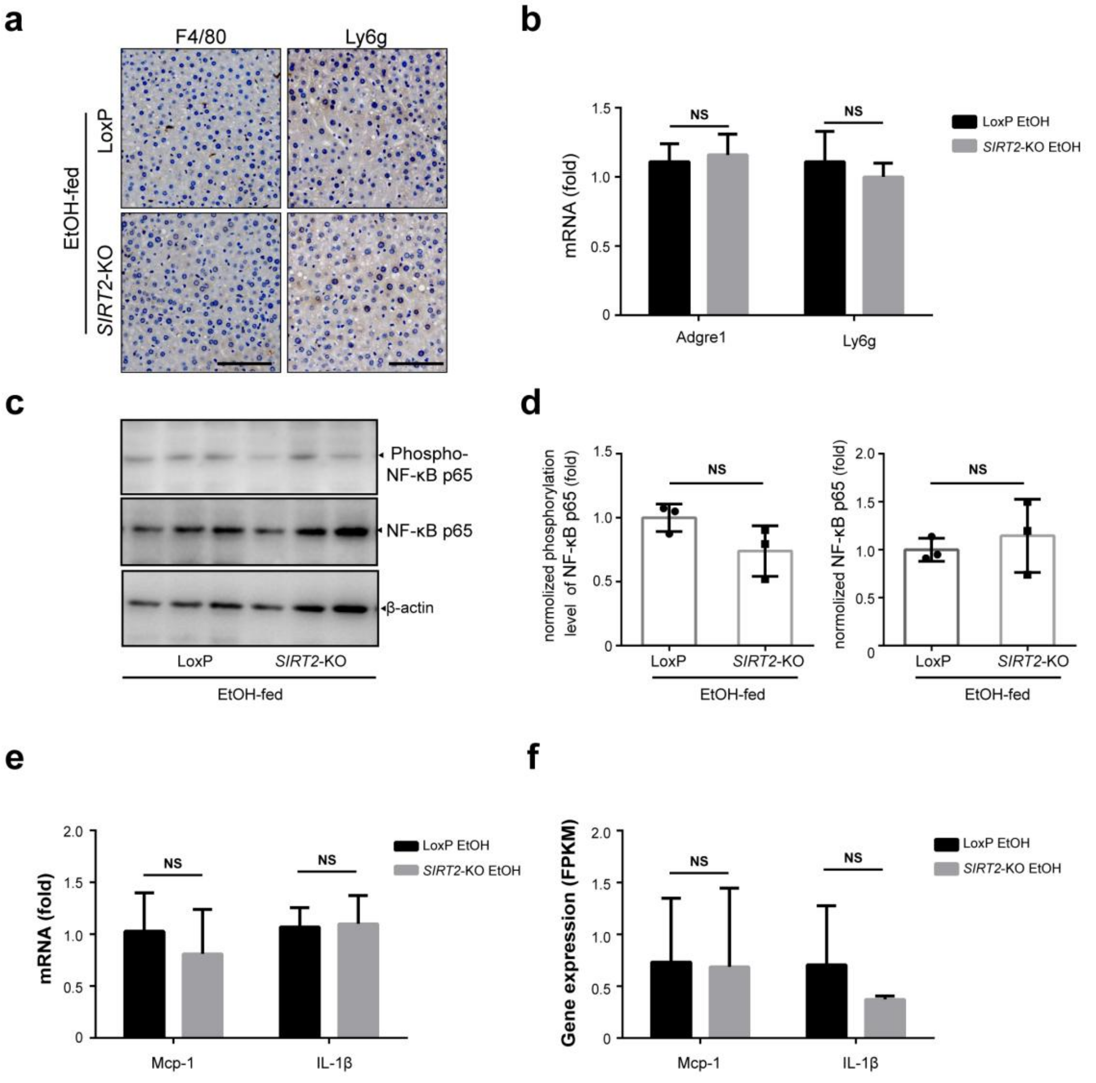

Supplementary Fig. S14. Liver-specific *SIRT2* deficiency has no effect on alcohol-induced inflammation in mice. a IHC detection of F4/80 and Ly6g (scale bar, 100 μm) in LoxP and *SIRT2*-KO mice treated with NIAAA model. **b** qRT-PCR analysis of *Adgre1* (a macrophage marker) and *Ly6g* (a neutrophil marker).**c** western blot analysis of phospho-NF-κB p65 and total NF-κB p65 expression in the murine livers. **d** The quantitative analysis of the phosphorylation levels of NF-κB p65 (phospho-NF-κB p65/total NF-κB p65, left) and the protein levels of NF-κB p65 in liver tissues (NF-κB p65/β-actin, right) by gray scanning. **e** qRT-PCR analysis of *Mcp-1* and *IL-1β*, **f** Fragments per kilobase of exon per million fragments mapped (FPKM) of *Mcp-1* and *IL-1β* by RNA-seq.

Supplementary tables

Supplementary Table S1. Characteristics of the normal controls and ALD patients.

Variables	Normal controls (n=12)	ALD patients (n=102)	*p* Value
**Age (years)**	54±5	50±10	0.16
**Gender-male (n,%)**	10(83)	84(82)	
**Total drinks in last 30 days**	0	204±52	$0.01*10^{-23}$
**Years of excessive drinking**	0	30±10	$0.03*10^{-16}$
**AST (U/L)**	16.5±4.6	55.61±52.16	0.01
**ALT (U/L)**	16.9±2.7	52.78±49.20	0.01
**Total bilirubin (mg/dl)**	0.46±0.15	1.09±1.69	0.22
**ALB (g/L)**	42.5±2.8	37.4±6.6	0.01
**Total protein (g/L)**	69.0±3.7	63.2±6.5	0.0033
**HB (g/L)**	131.5±7.8	118.3±24.7	0.14
**PLT (10^9/µl)**	197±25.9	191.8±94.1	0.81
**WBC counts (10^9/µl))**	6.6±0.8	8.5±6.9	0.33
**NEUT%**	62.7±3.8	62.8±16.6	0.99
**MONO%**	5.0±1.2	9.4±3	$0.21*10^{-5}$
**LYMPH%**	32.3±3.6	27.8±14.8	0.31
**Neutrophil counts (10^9/µl)**	4.1±0.6	5.5±4.5	0.29
**Monocyte counts (10^9/µl)**	0.3±0.1	0.75±0.73	0.05
**Lymphocyte counts (10^9/µl)**	2.1±0.4	2.3±4.5	0.92

ALD, alcoholic liver disease patients, WBC, white blood cells; HB, hemoglobin; PLT, platelet; AST, aspartate transaminase; ALT, alanine transaminase; ALB, albumin; LYMP%, percentage of lymphocytes to WBC; MONO%, percentage of monocytes to WBC; NEUT%, percentage of neutrophlis to WBC.

Supplementary Table S2. Characteristics of the ALD patients.

Variables	Patients with ALD (n=102)		*p* Value
	**SIRT2^{low}** (IHC score ≤ 5,n=47)	**SIRT2^{high}** (IHC score > 5,n=55)	
**Age (years)**	46±9	52±10	0.006
**Gender-male (n, %)**	35(74)	49(89)	
**Total drinks in last 30 days**	203±52	205±52	0.82
**Years of excessive drinking**	28±9	31±10	0.173
**AST (U/L)**	69.39±69.53	43.42±23.83	0.013
**ALT (U/L)**	65.70±65.88	41.57±21.86	0.011
**Total bilirubin (mg/dl)**	1.28±2.18	0.93±1.07	0.296
**ALB (g/L)**	37.4±6.8	37.2±6.5	0.88
**Total protein (g/L)**	63.2±6.6	63.1±6.5	0.9
**HB (g/L)**	118.6±25.8	122.2±22.8	0.464
**PLT counts (10^9/μl)**	219.6±101.1	173.2±83.2	0.013
**WBC counts (10^9/μl))**	9.7±8.4	7.5±5.1	0.109
**NEUT%**	64.8±17.3	61.1±16.1	0.272
**MONO%**	9.1±3.2	9.7±2.8	0.315
**LYMPH%**	26.1±15.2	29.1±14.5	0.256
**Neutrophil counts (10^9/μl)**	6.0±3.8	5.1±5.0	0.323
**Monocyte counts (10^9/μl)**	0.8±1.0	0.66±0.31	0.211
**Lymphocyte counts (10^9/μl)**	1.9±1.6	1.7±0.8	0.206

Supplementary Table S3. Antibodies used in this study.

Protein targets	Assays	Supplier	Cat no.
**Primary antibodys**			
SIRT2	WB/Co-IP/IF/IHC	Sigma	S8447
C/EBPβ	WB/IHC	Santa Cruz	sc7962
C/EBPβ	WB/IF	Abcam	ab32358
C/EBPβ-agarose	IP/ Co-IP	Santa Cruz	sc7962AC
LCN2	WB/IHC	Abcam	ab63929
cleaved-caspase3	WB/IHC	Cell Signaling Technology	9661
PARP-1	WB	Cell Signaling Technology	9532
Anti-acetyllysine rabbit pAb	IP	PTM BioLabs	PTM-105
Flag	WB/IP	Sigma	F1804
M2-beads	IP	Millipore	F2426
HA	IP	Sigma	H9658
Ubiqutin	IP	Cell Signaling Technology	3933
Mouse anti-4-Hydroxynonenal antibody	IHC	Alpha Diagnostic international	HNE 11-S
β-tubulin	WB	Sigma Cruz	T4026
Laminb	WB	Santa Cruz	sc6216

actin-HRP	WB	MBL	PM053-7
Phospho-NF-κB p65	WB	Cell Signaling Technology	3031S
NF-κB p65	WB	Santa Cruz	sc372
**Secondary antibodys**			
anti-rabbit IgG	WB	Cell Signaling Technology	7074V
anti-mouse IgG	WB	Cell Signaling Technology	7076V
anti-goat IgG	WB	Millipore	AP106P
FITC anti-rabbit IgG	IF	Santa Cruz	sc2365
Texas-Red anti-mouse IgG	IF	Abcam	ab6787

WB,western blot; Co-IP, co-immunoprecipitation ; IP, immunoprecipitation; IF, immunofluorescence; IHC, immunohistochemistry.

Supplementary Table S4. The cDNA target sequences of shRNAs, siRNAs, and primer sequences used in this study.

Name	Species	Sequence (5'->3')
**The cDNA target sequences of shRNAs and siRNAs**		
shSIRT2	Mouse	CCAACCATCTGCCACTACT
siSIRT2	Mouse	Forward:<br>GAAACAUCCGGAACCCUUCTT<br>Reverse:<br>GAAGGGUUCCGGAUGUUUCTT
siC/EBPβ	Mouse	Forward:<br>CCCUGCGGAACUUGUUCAATT<br>Reverse:<br>UUGAACAAGUUCCGCAGGGTT
**Primers sequences used for cloning**		
C/EBPβ	Mouse	Forward:<br>ATGGAAGTGGCCAACTTCTACTACG<br>Reverse:<br>CTAGCAGTGGCCCGCCGA
LCN2	Mouse	Forward:<br>ATGGCCCTGAGTGTCATGTGTC<br>Reverse:<br>TCAGTTGTCAATGCATTGGTCG

Primers sequences used for real-time PCR

Rpl13a	Mouse	Forward:
		GAGGTCGGGTGGAAGTACCA
		Reverse:
		TGCATCTTGGCCTTTTCCTT
SIRT2	Mouse	Forward:
		ATCGTGTTTTTCGGTGAGAACC
		Reverse:
		TTCCTTGTTAATGAGCAGCCG
Saa3	Mouse	Forward:
		TGCCATCATTCTTTGCATCTTGA
		Reverse:
		CCGTGAACTTCTGAACAGCCT
Saa2	Mouse	Forward:
		TGGCTGGAAAGATGGAGACAA
		Reverse:
		AAAGCTCTCTCTTGCATCACTG
Saa1	Mouse	Forward:
		CCCAGGAGACACCAGGATGAA
		Reverse:
		CCCTTGGAAAGCCTCGTGAA
Hpx	Mouse	Forward:

		AGCAGTGGCGCTAAATATCCT
		Reverse:
		CAACTCTCCCGTTGGCAGTA
Hp	Mouse	Forward:
		GCTATGTGGAGCACTTGGTTC
		Reverse:
		CACCCATTGCTTCTCGTCGTT
Orm2	Mouse	Forward:
		GTGTCCTAAGCATGGCACTG
		Reverse:
		GACACAGTGGTCATCTATGGTGT
Orm1	Mouse	Forward:
		TACAGGCAGGCAATTCAAACA
		Reverse:
		CCGAAGCTCTATTGTGTCGTTTA
Fga	Mouse	Forward:
		CACCTGCCTCATCTTGAGCG
		Reverse:
		GCATTGACTCTGATGTCTCTCCA
Fgb	Mouse	Forward:
		GGGAGTGTTGTGTCCTACGG
		Reverse:

		GCAATACTACTCTTGATTGGCCT
Fgg	Mouse	Forward:
		TTTCCTGTCTTCTTACCAAACCG
		Reverse:
		TGGTCTGGGTTGTAGTAAACCT
Fn1	Mouse	Forward:
		ATGTGGACCCCTCCTGATAGT
		Reverse:
		GCCCAGTGATTTCAGCAAAGG
LCN2	Mouse	Forward:
		TGGCCCTGAGTGTCATGTG
		Reverse:
		CTCTTGTAGCTCATAGATGGTGC
C/EBPβ	Mouse	Forward:
		GCCCGTTGCCAGGCG
		Reverse:
		TGGCCACTTCCATGGGTCTA
PTGS2	Mouse	Forward:
		TGAGCAACTATTCCAAACCAGC
		Reverse:
		GCACGTAGTCTTCGATCACTATC
CYP2E1	Mouse	Forward:

		CGTTGCCTTGCTTGTCTGGA
		Reverse:
		AAGAAAGGAATTGGGAAAGGTCC
Adgre1	Mouse	Forward:
		CTGCACCTGTAAACGAGGCTT
		Reverse:
		GCAGACTGAGTTAGGACCACAA
Ly6g	Mouse	Forward:
		GACTTCCTGCAACACAACTACC
		Reverse:
		ACAGCATTACCAGTGATCTCAGT
Mcp1	Mouse	Forward:
		TTAAAAACCTGGATCGGAACCAA
		Reverse:
		GCATTAGCTTCAGATTTACGGGT
Il1β	Mouse	Forward:
		TCAGGCAGGCAGTATCACTCA
		Reverse:
		TGCAGTTGTCTAATGGGAACGT
**Primer sequences used for ChIP**		
C/EBPβ binding primer I	Mouse	Forward:
		TCAGTAACAGCCTGGATCACAC

		Reverse:<br>ACATCGGCATGCAACACCAG
C/EBPβ binding primer II	Mouse	Forward:<br>CTGAGCCATCTCTCCAGTCC<br>Reverse:<br>AAGCTGTGTCGCCTTTGAAC
C/EBPβ binding primer III	Mouse	Forward:<br>TCCCCTGCTCAAGGTTATGG<br>Reverse:<br>ACGGAGGATAAGGGGATCACT
C/EBPβ binding primer Ⅳ	Mouse	Forward:<br>CACACCCACTTTCCCCAAGG<br>Reverse:<br>AAGGTAAGGACTGCAACCTCG
C/EBPβ binding primer Ⅴ	Mouse	Forward:<br>TGGGAATGTCCCTCTGGTCC<br>Reverse:<br>CCAGGTCGGAACTGTGCAA
C/EBPβ binding primer VI-VIII	Mouse	Forward:<br>CTTCCTGTTGCTCAACCTTGC<br>Reverse:<br>GGCGGGGTAGTCCATCCTTT

Distant region primer	Mouse	Forward:<br>TGGGGGCTCAGCTATTTGTG<br>Reverse:<br>GCAGCCAGCCTACTACAAGT
PPARγ primer	Mouse	Forward:<br>ACTGGCGAGACAATGTAGCA<br>Reverse:<br>CCTGACTGAGAGCCAGTTGT

ChIP, chromatin immunoprecipitation.
